# Association between surgeon volume and patient outcomes after elective shoulder replacement surgery using data from the National Joint Registry and Hospital Episode Statistics for England: population based cohort study

**DOI:** 10.1136/bmj-2023-075355

**Published:** 2023-06-21

**Authors:** Epaminondas Markos Valsamis, Gary S Collins, Rafael Pinedo-Villanueva, Michael R Whitehouse, Amar Rangan, Adrian Sayers, Jonathan L Rees

**Affiliations:** 1 Nuffield Department of Orthopaedics, Rheumatology and Musculoskeletal Sciences, Botnar Research Centre, University of Oxford, Oxford OX3 7LD, UK; 2 NIHR Oxford Biomedical Research Centre, Oxford, UK; 3 Nuffield Department of Orthopaedics, Rheumatology and Musculoskeletal Sciences, Centre for Statistics in Medicine, University of Oxford, Oxford, UK; 4 Musculoskeletal Research Unit, Bristol Medical School, Southmead Hospital, University of Bristol, Bristol, UK; 5 NIHR Bristol Biomedical Research Centre, Bristol, UK; 6 Health Sciences and Medical School, University of York, York, UK

## Abstract

**Objective:**

To investigate the association between surgeon volume and patient outcomes after elective shoulder replacement surgery to improve patient outcomes and inform future resource planning for joint replacement surgery.

**Design:**

Prospective cohort study.

**Setting:**

Public and private hospitals in the United Kingdom, 2012-20.

**Participants:**

Adults aged 18 years or older who had shoulder replacement surgery, identified in the National Joint Registry, with linkage of participants in England to Hospital Episode Statistics data.

**Main outcome measures:**

The main outcome measure was revision surgery. Secondary outcome measures were reoperation within 12 months, serious adverse events, and prolonged hospital stay (>3 nights) after shoulder replacement surgery.

**Results:**

39 281 shoulder replacement procedures undertaken by 638 consultant surgeons at 416 surgical units met the inclusion criteria and were available for analysis. Multilevel mixed effects models and restricted cubic splines were fit to examine the association between a surgeon’s mean annual volume and risk of adverse patient outcomes, with a minimum volume threshold of 10.4 procedures yearly identified. Below this threshold the risk of revision surgery was significantly increased, as much as twice that of surgeons with the lowest risk (hazard ratio 1.94, 95% confidence interval 1.27 to 2.97). A greater mean annual surgical volume was also associated with a significantly lower risk of reoperations, fewer serious adverse events, and shorter hospital stay, with no thresholds identified. Annual variation in surgeon volume was not associated with any of the outcomes assessed.

**Conclusions:**

In the healthcare system represented by these registry data, an association was found between surgeons who averaged more than 10.4 shoulder replacements yearly and lower rates of revision surgery and reoperation, lower risk of serious adverse events, and shorter hospital stays. These findings should inform resource planning for surgical services and joint replacement surgery waiting lists and improve patient outcomes after shoulder replacement surgery.

## Introduction

Shoulder replacement is an established surgical treatment option for end stage shoulder arthritis.^1^ The global incidence of shoulder replacements is rising rapidly, with some countries reporting up to a 17-fold increase over the past 10 years.^2^ More than 8000 shoulder replacements are performed annually in the United Kingdom alone, with numbers continuing to escalate.^3^ ^4^ With changes in population demographics (age, sex, and obesity), the incidence of joint replacements is forecast to increase, with projections for shoulder replacement rates outpacing even those of hip and knee replacements.^5^ ^6^

The longer waiting times for elective surgery after the covid-19 pandemic have been exacerbated by staff shortages and a lack of capacity in healthcare systems such as the National Health Service.^7^ Early projections from the National Joint Registry based on the first lockdown in 2020 estimated that it will take five years to clear the backlog of patients waiting for surgery—and that estimate is more likely to be 10 years if data from subsequent lockdowns and the low surgery rates of 2021 are also considered.^8^ Despite orthopaedic services dominating elective surgery numbers in most hospital units, surgery for patients with musculoskeletal pain and disability is not always considered urgent and so such surgeries have been particularly affected by the pandemic while others have been prioritised.^9^ ^10^ Previous major reconfiguration of specialist care across the UK, such as cancer services and major trauma networks, have contributed to improved patient survival.^11^ Evidence suggests that increased surgeon and surgical unit volume are associated with improved patient outcomes across surgical specialties and healthcare systems.^12^ ^13^ Early results for hip and knee replacements have shown reduced revision rates and hospital stays in high volume centres, and therefore considerable cost savings.^14^ Surgeon volume is also receiving more attention, with studies showing better patient outcomes if joint replacement is performed by surgeons with at least a minimum threshold number of procedures yearly.^15^ ^16^ ^17^

Improving patient outcomes by avoiding repeat (revision) surgery, complications, and prolonged hospital stays is crucial to reduce the burden and cost to healthcare systems. In the UK, the Department of Health and Social Care and NHS England recently highlighted the need to accelerate the development of the NHS’s Getting It Right First Time (GIRFT) surgical transformation programme to aid recovery in services, with a focus on the implementation of surgeon and surgical unit case load requirements that improve patient outcomes at a reduced cost.^18^ Despite the increase in shoulder replacement surgery, evidence to guide any such service provision planning remains insufficient.^15^ ^19^ We therefore evaluated the association between surgeon volume and patient outcomes after shoulder replacement surgery with a view to further improve patient outcomes and help inform any national healthcare resource planning around joint replacement surgery.

## Methods

### Study design and data sources

In this population based cohort study we used routinely collected hospital and joint registry data in the UK from 1 April 2012 to 31 December 2020. All shoulder replacements recorded by the National Joint Registry of England, Wales, Northern Ireland, the Isle of Man, and Guernsey were considered for the study. Records for England were linked to the Hospital Episode Statistics Admitted Patient Care database managed by NHS Digital. Data submission to the National Joint Registry is mandatory for all shoulder replacement procedures in the public and private sectors in the areas covered by the registry and include detailed data on patients, surgeons, and operations. The Hospital Episode Statistics Admitted Patient Care database records all inpatient and day case activity carried out by NHS hospitals and NHS funded care in England and contains demographic data, medical diagnoses, and procedural and administrative information. Hospital Episode Statistics data are used to ensure accurate remuneration to NHS providers for their activities. Data were linked to the Civil Registration Mortality database.

We used unlinked National Joint Registry data to analyse the effect of surgeon volume on revision surgery. Linked National Joint Registry and Hospital Episode Statistics data were used to analyse the effect of surgeon volume on serious adverse events, reoperations, and prolonged hospital stay (>3 nights).

### Selection criteria

All consenting adults aged 18 years or older who had a primary shoulder replacement were eligible for inclusion in the study. We included patients if their surgical history was consistent (ie, their date of revision or death did not pre-date their primary surgery) and did not contain duplicates. Calculations of surgeon volume took into account shoulder replacement surgery for both acute trauma and elective indications, capturing all shoulder replacement activity contributing to surgeon experience. However, patients who require surgery for acute trauma represent a distinct cohort with more variable outcomes arising from their unique presentation and associated injuries, so we only included procedures for elective indications in subsequent analysis. We included patients if the primary surgeon or assistant surgeon for their procedure was a consultant, and we excluded patients when the main surgical indication was malignancy. We also excluded patients scheduled for surgery with a consultant who had fewer than 365 days of National Joint Registry data, as we were unable to calculate the surgeon’s annual volume.

### Primary outcome

The National Joint Registry defines revision surgery as a procedure that involves adding, removing, or modifying one or more components of a joint prosthesis and is the most universally accepted outcome for evaluating joint replacement surgery.^3^ Patients were censored after death and data were reformatted to include time to event variables in a format suitable for survival analysis. As the estimand for our primary outcome was the net failure of the implant, we used the Kaplan-Meier estimator.^20^ ^21^ ^22^

### Secondary outcomes

Some patients who have a primary shoulder replacement subsequently undergo a different type of shoulder procedure on the same side, which does not involve changing the shoulder replacement implants. These procedures are therefore not considered revisions, but they are reoperations and it is important to record such procedures. To capture all subsequent surgery that is relevant to the primary shoulder replacement, we recorded any of the following reoperations on the same shoulder occurring within 12 months of the index procedure, and on a separate occasion to any revision surgery: subacromial decompression or acromioclavicular joint excision, or both; rotator cuff repair; manipulation under anaesthesia or release, or both; washout or debridement; synovectomy; osteomyelitis surgery; complex reconstruction; bone resection; arthroscopy or surgery for instability; reduction of dislocation; or fixation of periprosthetic fracture. Serious adverse events were defined as medical complications severe enough to require admission to hospital, including pulmonary embolism, myocardial infarction, lower respiratory tract infection, acute kidney injury, urinary tract infection, cerebrovascular events, and all cause death.^23^ Serious adverse events were identified using ICD-10 (international classification of diseases, 10th revision) codes from Hospital Episode Statistics data and categorised into those occurring within 30 days or 90 days of the index procedure. We defined prolonged hospital stay as an inpatient duration greater than three nights from the date of the index procedure. Reoperations, 30 day and 90 day serious adverse events, and prolonged hospital stay were treated as binary variables representing the presence of the event.

### Primary independent variable

The primary independent variable of interest was consultant surgical volume in the 365 days before the primary shoulder replacement so surgeon volume could change dynamically over time. The primary independent variable was calculated before excluding patients for the surgical indication of acute trauma, and consultant surgical volume encompassed all primary shoulder replacement surgery that met the selection criteria.

### Confounding factors

Models were adjusted for several covariates that have been shown to affect the risk of revision and complications. Confounding factors were selected a priori and organised into four groups: patient factors (age, sex, American Society of Anaesthesiologists grade, indication for surgery, and previous shoulder surgery), operation factors (surgical approach, type of anaesthetic, thromboprophylaxis, year of surgery, and type of shoulder replacement procedure), centre factors (setting of treatment (private or NHS hospital) and surgical unit volume), and consultant factors (training status of the primary surgeon performing the surgery and that of the assistant surgeon, and whether the consultant was considered as newly registered, defined as less than five years of activity in the National Joint Registry that started at least one year after the start of data collection).

### Statistical analysis

To enable evaluation of both the mean surgical volume and the annual variation in volume, we used a process known as group mean centring whereby two new variables are generated from the primary independent variable.^24^ ^25^ The first variable, mean annual consultant volume, was the mean of the primary independent variable across all procedures undertaken by a particular consultant. The second variable, deviation annual consultant volume, was the difference between the primary independent variable for each procedure (ie, consultant surgical volume in the 365 days before the primary shoulder replacement) and the mean annual consultant volume. We examined the distributions of these variables by plotting histograms and empirical cumulative frequency distributions. The supplementary file shows an example of the group mean centring process.

Continuous variables, including surgeon and unit volume, were modelled with restricted cubic splines to allow for non-linear effects. The Akaike Information Criterion was used to select the most parsimonious specification of restricted cubic splines using unadjusted models.^26^ The multilevel model framework accounts for the clustered structure of surgical data where patient level data are nested within the surgeon undertaking their procedure.^27^ A multilevel mixed effects parametric (Weibull) survival model was used for revision, whereas multilevel mixed effects logistic models were used for reoperations, serious adverse events, and prolonged hospital stay using the *mestreg* and *melogit* packages, respectively, in Stata version.16.1.^28^ Adjustment for confounding was undertaken incrementally, adjusting for each of the four groups of confounding variables to explore their influence on the volume effect at each stage. The supplementary file provides details of the model specifications. If we observed an inflection point in the association between surgeon volume and risk of each outcome, differentiation was used to determine the local minimum. The mean annual consultant volume was centred on this value to calculate the surgical volume threshold below which patients are at a significantly increased risk of that outcome.

We excluded 338 patients from the National Joint Registry dataset who had no recorded outcome, representing just 0.6% of all patients, and undertook a complete case analysis. Data were not missing for other variables used in the primary analysis.

#### Sensitivity analysis

The linked National Joint Registry and Hospital Episode Statistics data contained potentially important information on patients’ characteristics that was not available from the unlinked National Joint Registry data alone. To investigate the potential impact on the results of including ethnic group, region of treatment, and deprivation (index of multiple deprivation), we additionally adjusted for these covariates in a sensitivity analysis of the secondary outcomes. As ethnic group and index of multiple deprivation were missing in 9.2% and 1.3% of records, respectively, we performed multiple imputation by chained equations. Rubin’s rules were used to pool the results from the multilevel models for each of 10 imputed datasets.

### Patient and public involvement

Several of the top 10 research uncertainties from the 2015 James Lind Alliance Priority Setting Partnership on shoulder surgery related to shoulder replacements.^29^ Patient representatives sit on the committee structure of the National Joint Registry and on a patient advisory panel for this study. While outcomes and revision surgery after shoulder replacement surgery are important to patients, our patient representatives also thought that hospital stay was an important factor to consider in this study, and that an inpatient stay greater than three nights was excessive and should be considered as prolonged. Patient representatives endorsed the growing importance of surgeon and hospital volume, as the NHS Constitution gives most people living in England the right to choose where to receive treatment, and such metrics are now publicly available through the National Joint Registry.^30^ ^31^

## Results

### Patients, characteristics, and distributions

**Fig 1:**
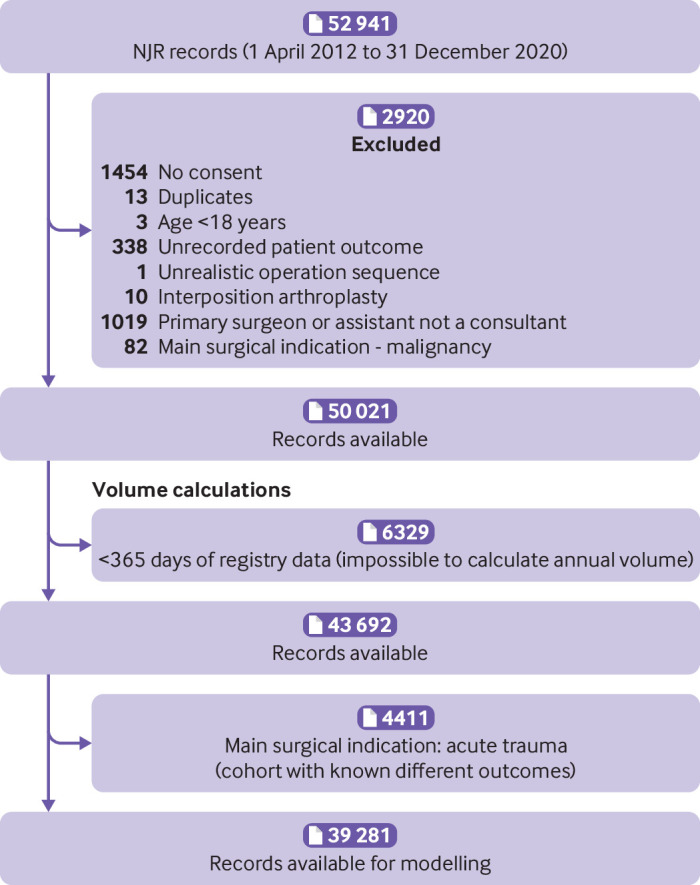
Flowchart for unlinked National Joint Registry (NJR) dataset used for primary outcome of revision surgery after shoulder replacement surgery

**Fig 2:**
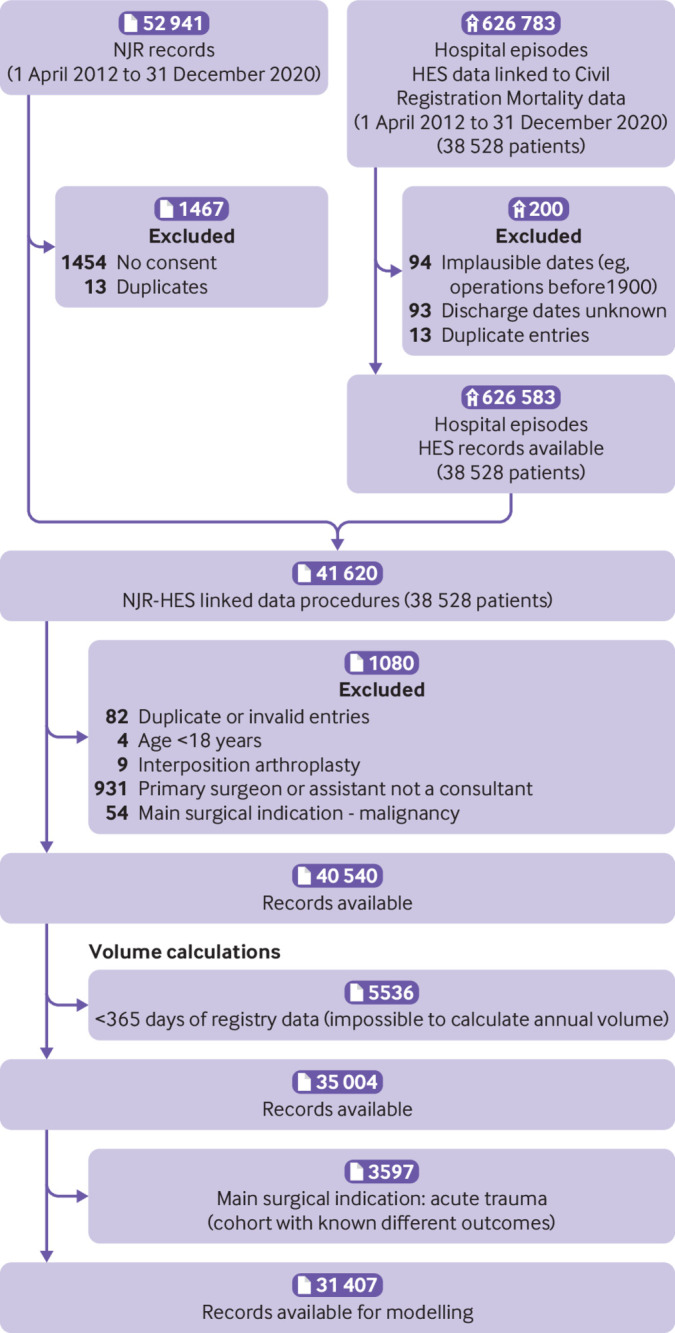
Flowchart for linked National Joint Registry (NJR) and Hospital Episodes Statistics (HES) dataset used for secondary outcomes of reoperation within 12 months, serious adverse events, and prolonged hospital stay after shoulder replacement surgery

A total of 52 941 shoulder replacement procedures were recorded in the National Joint Registry between 1 April 2012 and 31 December 2020 (fig 1). After applying the eligibility criteria, 39 281 shoulder replacement procedures were available in the unlinked National Joint Registry dataset for analysis of revision surgery, including 1379 revisions, with a maximum follow-up of 7.75 years and a total of 147 802 years of observation time. These procedures were performed by 638 consultant surgeons at 416 surgical units. The number of procedures available for the analysis of reoperations, serious adverse events, and prolonged hospital stay in the linked National Joint Registry and Hospital Episode Statistics dataset were 31 407 (fig 2). Hospital Episode Statistics does not contain data from procedures that are performed in private hospitals not funded by the NHS and in hospitals outside England. The supplementary file provides descriptive statistics for the population cohorts from each dataset, including summary statistics of surgeon volume by each confounding factor.

**Fig 3:**
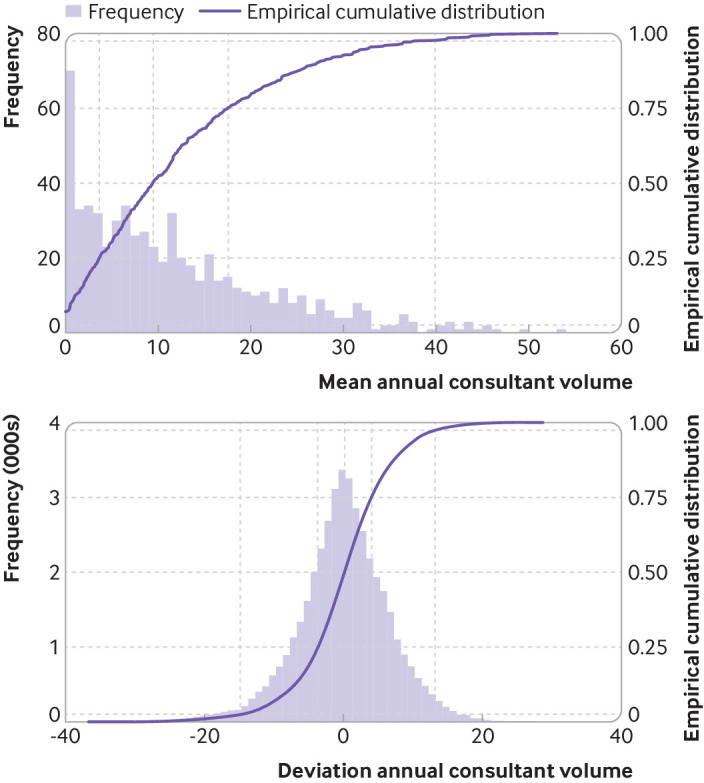
Distributions of mean annual consultant volume and deviation annual consultant volume. Dashed lines denote 2.5, 25, 50, 75, 97.5 centiles. Mean annual consultant volume is a consultant specific variable, whereas deviation annual consultant volume is a procedure specific variable

The mean annual consultant volume from the unlinked National Joint Registry dataset was positively skewed, with a median of 9.4 and less than 2.5% of surgeons undertaking more than 40 procedures yearly. Overall, 70 surgeons undertook less than one procedure yearly on average (mean annual consultant volume <1), of which 46 surgeons never undertook more than one procedure within the same year (mean annual consultant volume equal to zero). The deviation annual consultant volume was naturally centred at zero, with around 50% of procedures having a deviation less than ±4, and around 5% of procedures having a deviation greater than ±14 (fig 3). Higher volume surgeons were more likely to undertake a reverse total shoulder replacement, use the Superior (MacKenzie) surgical approach, and not use regional anaesthesia. They were more likely to be experienced consultants working at a higher volume surgical unit and to be assisting the primary surgeon (see supplementary file).

**Fig 4:**
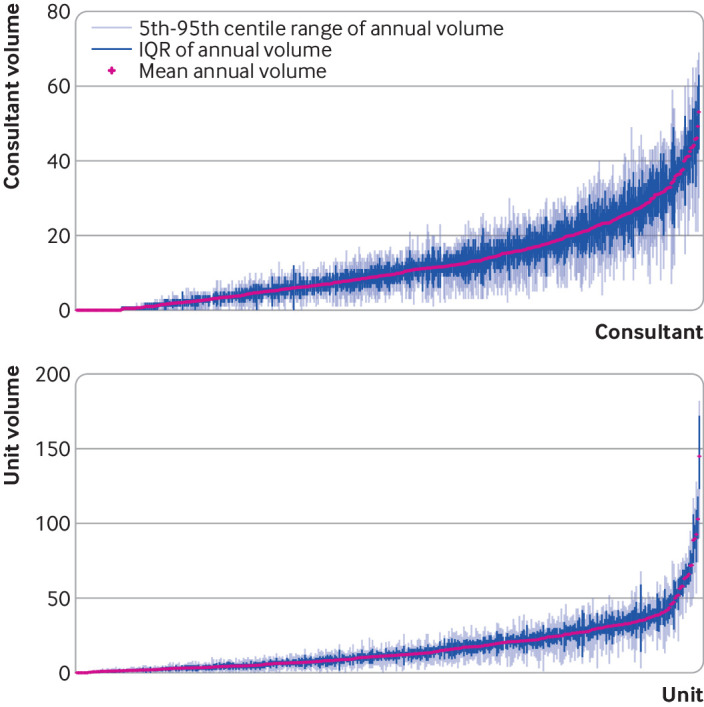
Consultant surgeon and surgical unit ranked mean annual volume with interquartile range (IQR) and 5th-95th centile range of volume distributions plotted

Surgeons and units with greater mean annual volumes had greater variability in operating volume, as would be expected (fig 4). The interquartile range was more negatively skewed for surgical units compared with surgeons. The median interquartile range of volume in the past 365 days for surgeons was 4 (SD 4.5) procedures yearly, whereas that for surgical units was 6 (SD 6.3), showing greater variability for surgical units. Although some high volume surgeons were outliers, the highest volume surgical units appeared to deviate more noticeably.

### Primary outcome: Revision

**Fig 5:**
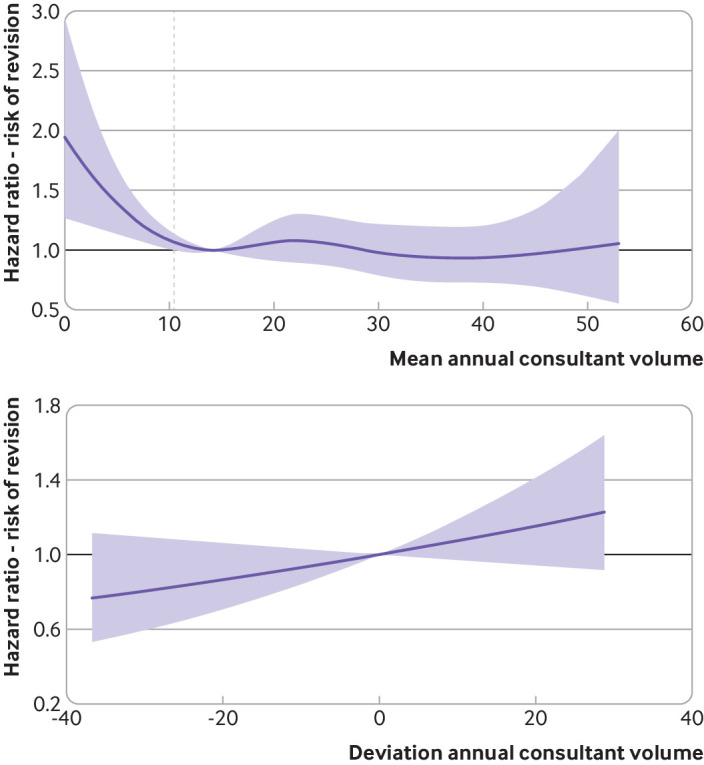
Association of mean annual consultant volume and deviation annual consultant volume on risk of revision adjusted for confounding factors in a multilevel parametric survival model. Mean annual consultant volume represents the mean of the primary independent variable across all procedures undertaken by a particular consultant. Deviation annual consultant volume represents the difference between the primary independent variable for each procedure and the mean annual consultant volume. Shaded areas represent 95% confidence intervals. Vertical dashed line represents the threshold of 10.4

The risk of revision decreased with increasing mean annual consultant volume, before reaching a plateau (fig 5). Centred on the local minimum inflection point, where the plateau is attained (14.3) for the fully adjusted model, the hazard ratio of revision was significantly greater for surgeons with a mean annual consultant volume of less than 10.4, whereas for volumes greater than 10.4 the reduction in revision risk with increased volume was not significant. The reduction in revision risk from 0 (representing a surgical frequency less than once yearly) to 10.4 procedures was 44.8% (hazard ratio 1.94 (95% confidence interval 1.27 to 2.97) to 1.07 (1.00 to 1.14)). The effect of confounding adjustment was small, with a standard deviation of 0.43 for the minimum volume threshold for incremental levels of confounding adjustment.

No effect of the deviation annual consultant volume on risk of revision (the confidence intervals crossed the value of 1) was observed, with adjustment for confounding having minimal effect on the deviation annual consultant volume (see supplementary file).

### Secondary outcomes: Reoperations, serious adverse events, and prolonged hospital stay

**Fig 6:**
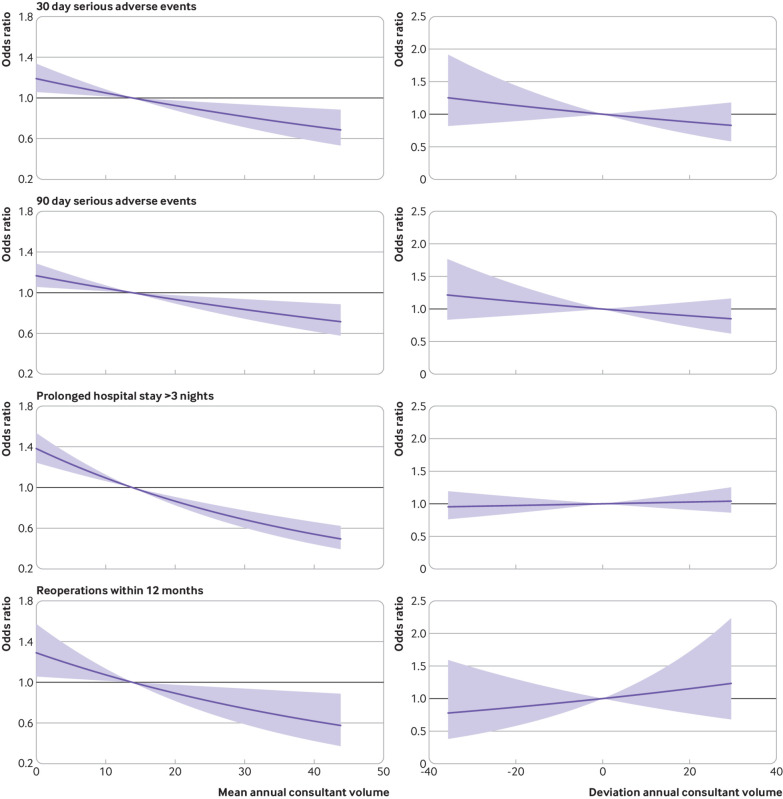
Association of mean annual consultant volume and deviation annual consultant volume on risk of serious adverse events (30 day and 90 day), prolonged hospital stay (>3 nights), and reoperation, adjusted for confounding factors in multilevel logistic regression models. Shaded areas represent 95% confidence intervals

The observed effect of mean annual consultant volume and deviation annual consultant volume on risk of reoperations, serious adverse events, and prolonged hospital stay were similar. An increased mean annual consultant volume was associated with a near linear decrease in the risk of these events, whereas the effect of the deviation annual consultant volume was not significant (fig 6).

The effect of the mean annual consultant volume showed no evidence of a plateau on any of these outcomes, although the effect on prolonged hospital stays and on reoperations appeared to occur at a decreasing rate. The greatest effect of mean annual consultant volume was for prolonged hospital stay, with a relative reduction of 61.9% (odds ratio 1.38 (95% confidence interval 1.24 to 1.54) to 0.53 (0.43 to 0.65)) between the 2.5th and 97.5th centiles of volume (0 and 40 operations). Comparatively, the relative risk reduction was 40.4% (1.19 (1.06 to 1.34) to 0.71 (0.56 to 0.89)) for 30 day serious adverse events, 36.9% (1.17 (1.06 to 1.29) to 0.74 (0.61 to 0.90)) for 90 day serious adverse events, and 53.1% (1.29 (1.06 to 1.57) to 0.60 (0.41 to 0.90)) for reoperation.

The observed effect of confounding adjustment was most noticeable for 30 day serious adverse events where the crude model showed no significant association, whereas the effect on reoperation was the least noticeable (see supplementary file).

### Sensitivity analysis

Additional adjustment for ethnic group, region of treatment, and deprivation resulted in only a small difference to the observed relative risk reduction for each secondary outcome (see supplementary file for detailed analyses). When adjusting for all additional covariates together, the relative risk reduction was 49.1% (odds ratio 1.25 (95% confidence interval 1.15 to 1.36) to 0.64 (0.43 to 0.85)) for prolonged hospital stay, 35.9% (1.16 (1.04 to 1.28) to 0.74 (0.50 to 0.98)) for 30 day serious adverse events, 31.4% (1.13 (1.03 to 1.24) to 0.78 (0.58 to 0.98)) for 90 day serious adverse events, and 56.6% (1.32 (1.12 to 1.53) to 0.57 (0.17 to 0.98)) for reoperation. No significant effect was found for deviation annual consultant volume.

## Discussion

Data from the National Joint Registry and Hospital Episode Statistics in England were used to explore the association between surgeon volume and the primary outcome of revision and secondary outcomes of reoperation, serious adverse events, and prolonged hospital stay after elective shoulder replacement surgery in first time recipients using multilevel models. We found an association between consultant surgeons who averaged less than 10.4 procedures yearly and an increased risk of revision surgery. Patients who received shoulder replacements by higher volume surgeons showed a reduced risk of reoperation and serious adverse events and had a shorter hospital stay. Annual variation in surgeon volume did not appear to be associated with any of these outcomes.

### Strengths and weaknesses of this study

Apart from the large sample size, this study has several strengths. Firstly, we investigated the effect of surgeon volume on multiple patient outcomes, including some identified as most important to patients. This was made possible using linked national registry and hospital data. Secondly, the data used reflect the volume of surgery undertaken across different geographical regions, representing all the main types of shoulder replacement procedures and patients of different ages, ethnicity, and socioeconomic groups, providing a complete picture of shoulder replacement activity across a national healthcare system. Thirdly, the use of restricted cubic splines enabled the modelling of non-linear volume associations and the identification of the minimum volume threshold by centring on the local minimum inflection point. Fourthly, although most analyses used single level models and therefore assumed that all operations are independent of one another, the multilevel models used in this study more accurately reflect the clustered nature of surgical procedures, where surgeons usually operate on more than one patient. Fifthly, group mean centring enabled the analysis of the effect that any deviations in surgical volume may have on patient outcomes, addressing real life variations in procedure volume. Finally, extensive adjustment for confounding showed a relatively small influence on the volume effect. In the sensitivity analysis, the observed associations for the secondary outcomes were consistent when adjusting for additional patient demographics unavailable from the National Joint Registry.

Despite these strengths, this study has certain limitations. Firstly, owing to the differences in coverage of the national joint registry and hospital databases, procedures undertaken in private hospitals that were not funded by the NHS and in hospitals outside of England were not included in the analysis of reoperation, serious adverse events, or prolonged hospital stay. Secondly, while the mean annual and deviation surgical unit volumes were included as confounding variables, the interaction between patients, surgeons, and surgical units is likely to be considerably more complicated, with different units having a variable surgical workforce and surgeons working in more than one unit. It is therefore likely that the two-level models developed are a simplification of real life practice.^3^ Thirdly, length of hospital stay is likely to be confounded by social circumstances and carer availability, neither of which is captured by these databases. Hospital stay was, however, identified as an outcome of particular importance for patients and their families, especially considering the possibility that patients may need to travel further away for treatment if centralisation of shoulder replacement services was ever considered. Fourthly, we were unable to adjust for patients’ body mass index, which is a potential confounding factor. Body mass index is, however, strongly associated with other confounders that were adjusted for, such as American Society of Anaesthesiologists grade.^32^ Fifthly, although the dataset used was large, it was not large enough to reliably examine the effect on patient outcomes if a long term sustained change in a surgeon’s annual volume occurred (ie, an initially low volume surgeon subsequently became a high volume surgeon for several years). Finally, although the periodicity (annual) of the calculated volume variables adhered to convention and accommodated for any seasonal variation, it also meant that the volume of a newly starting consultant surgeon could not be measured until a year had elapsed, and therefore the analysis could not capture the outcomes for these first patients.

### Comparison with other studies

Other studies have shown evidence of an association between surgeon volume and outcome for shoulder replacements, but they were prone to methodological weaknesses such as arbitrary categorisation of volume, limited adjustment for confounding, and small sample sizes.^33^ ^34^ ^35^ One large study attempted to assess the association between surgeon volume and outcome for patients undergoing shoulder replacement using data from the Australian Orthopaedic Association National Joint Replacement Registry. The authors sought to investigate the effect of surgeon volume on risk of revision in 28 752 participants.^2^ Although they did identify differences in revision risk between surgeons averaging less than 10, 10-20, or more than 20 shoulder replacements yearly, they found this difference was significant for one type of shoulder replacement (reverse total shoulder replacement) when used for one type of arthritis over the study period, but the differences observed for other indications or for other shoulder replacement types (conventional anatomical shoulder replacement) persisted only for a few months after surgery. Although the results of that study provide evidence of a volume-outcome association, several methodological differences might explain the different conclusions—shoulder hemiarthroplasty replacement procedures were excluded, and adjustments were only made for age and sex. The study also arbitrarily categorised surgeons into three volume groups, whereas we considered volume as a continuous variable modelled using a multilevel framework. Indeed, categorising volume by arbitrarily assigning groups limits a study’s ability to delineate the nature of the volume-outcome association and so prevents reporting of minimum surgical volume thresholds.^36^ Studies using target trial emulation in other healthcare settings have identified the importance of an accurate specification of model parameters and may help guide future research on the association between volume and outcome.^37^

Group mean centring and multilevel models have been used successfully to investigate different aspects of surgical volume, and offer novel insights into the effect of surgeon volume on revision risk after hip replacement.^38^ One study found that about 200 mean annual hip replacements were required to attain a plateau for revision risk, and indeed, different surgical procedures seem to have noticeably different volume-outcome associations.^39^ This difference may be due to different learning curves across procedures and specialties but may also be due to the considerable difference in prevalence and absolute surgical volume between upper and lower limb joint replacement surgery.^40^

### Implications for clinicians and policy makers

The three main findings from this UK based healthcare study were that the average annual volume during a surgeon’s career seems to be more important than annual variation in volume; an association was observed between consultant surgeons who averaged less than 10.4 procedures yearly and an increased risk of revision surgery; and a near linear association exists between surgeon volume and adverse events after shoulder replacement surgery, with patients receiving shoulder replacements by higher volume surgeons showing a reduced risk of reoperation, fewer serious adverse events, and shorter hospital stay.

Improving outcomes and reducing complications after joint replacement surgery is of clear benefit to patients and their families, but the results of this study also provide timely evidence for healthcare systems that are overburdened, under-resourced, and in need of recovery planning after the covid-19 pandemic. During such times the risk is that healthcare providers and funders become overly focused on waiting list targets. This study offers evidence for local hospitals and national healthcare services that informs workforce and resource planning to ensure the best outcomes for patients undergoing shoulder replacement surgery. Besides providing evidence to reduce healthcare burden in the short term by minimising complications and hospital stay, this study provides information on annual surgical volumes that can help towards reducing the need for revision surgery in the medium to longer term.

### Unanswered questions and future research

Further research is needed to understand the impact on patient outcomes if a long term sustained change in a surgeon’s annual volume occurs. Although the results of this study address the role of individual surgeon volume on patient outcomes, further work is needed to fully understand the complex interaction between this and hospital volume, especially if it is to inform any discussions around the centralisation or redistribution of some treatments and services.

**What is already known on this topic**

Evidence suggests that surgeon volume is associated with improved patient outcomes across different procedures in different specialties and healthcare systems
Some national healthcare providers focus on the implementation of minimum volume thresholds for surgeons to improve patient outcomes (eg, hip and knee replacements)
Evidence is insufficient to guide any such service provision planning for shoulder replacement surgery

**What this study adds**

An association was observed between surgeons who averaged fewer than 10.4 shoulder replacement procedures yearly and increased revision rates
Annual variation in a surgeon’s volume compared with their career mean was not associated with any of the outcomes assessed
The lowest risk of reoperation and serious adverse events, including shorter hospital stays, were observed in patients who had shoulder replacements by high volume surgeons

## Data Availability

Access to the data analysed in this study required permission from the National Joint Registry research subcommittee. Information on research data access request to the National Joint Registry is available at https://www.njrcentre.org.uk/research/research-requests/.

## Web extra

Extra material supplied by authors

Supplementary information: Additional tables 1-9 and details of multilevel models

## Notes

The lead author (EMV) and manuscript’s guarantor, affirms that the manuscript is an honest, accurate and transparent account of the study being reported; that no important aspects of the study have been omitted; and that any discrepancies from the study as originally planned (and, if relevant, registered) have been explained.

Dissemination to participants and related patient and public communities: The National Joint Registry and its patient representative group have been made aware of the results of this study and will support the dissemination of results through their networks. Dissemination to the wider public will be supported by the University of Oxford and the Oxford National Institute for Health and Care Research Biomedical Research Centre through the press, media, and charities. Surgeons and policy makers will be informed of the results through presentations at national and international scientific meetings.

Provenance and peer review: Not commissioned; externally peer reviewed.
