# Supplementary material for: Association between surgeon volume and patient outcomes after elective shoulder replacement surgery using data from the National Joint Registry and Hospital Episode Statistics for England: population based cohort study
Source: BMJ. 2023 Jun 21;381:e075355. doi: 10.1136/bmj-2023-075355 (PMC10283034; doi:10.1136/bmj-2023-075355)
Supplement: Supplementary file 1 — Supplementary information: Additional tables 1-9 and details of multilevel models [file vale075355.ww.pdf]

Supplementary material

Contents

Table 1: Coding and specification of confounding variables 2

Table 2: Descriptive statistics of continuous variables 3

Table 3: Descriptive statistics of categorical variables 3

Table 4: 365-day consultant volume for each category of confounding variable: 5

Table 5: Descriptive statistics by mean annual consultant volume, categorised by 25th, 50th and 75th percentiles- patient age at primary: 7

Table 6: Descriptive statistics by mean annual consultant volume, categorised by 25th, 50th and 75th percentiles - categorical variables: 8

Table 7: Group mean centring: 10

Table 8: OPCS-4 codes to identify reoperations 10

Table 9: ICD-10 codes to identify serious adverse events 11

Multilevel models 12

Primary outcome (Revision): Multilevel (Weibull) survival model 12

Secondary outcomes (reoperations, serious adverse events, prolonged hospital stay): Multilevel logistic model 12

Incremental confounding adjustment for primary and secondary outcomes 13

Revision: 14

30-day serious adverse events (SAE): 15

90-day serious adverse events (SAE): 16

Prolonged hospital stay (>3 nights): 17

Reoperations: 18

Sensitivity analysis 19

Complete case analysis: adjusting for IMD only (n=30,991) 20

Complete case analysis: adjusting for ethnic group only (n=28,507) 21

Complete case analysis: adjusting for treatment region only (n=31,407) 22

Multiple imputation of ethnic group and IMD: adjusting for ethnic group only (n=31,407) 23

Multiple imputation of ethnic group and IMD: adjusting for all three additional covariates together (n=31,407) 24

Table 1: Coding and specification of confounding variables

Variable	Options	Regression specification
Age	Restricted cubic splines	Continuous
Sex	Male/Female	Binary
American Society of Anaesthesiologists (ASA) grade	1/2/3/4+5	MV binary
Main surgical indication	Avascular necrosis/Cuff tear arthropathy/Inflammatory arthropathy/Osteoarthritis/Other/Trauma sequelae	MV binary
Previous shoulder surgery	Yes/No	Binary
Surgical approach	Deltopectoral/Superior(MacKenzie)/Other	MV binary
General anaesthetic	Yes/No	Binary
Regional anaesthetic	Yes/No	Binary
Chemical thromboprophylaxis	Yes/No	Binary
Mechanical thromboprophylaxis	Yes/No	Binary
Year of surgery	2013/2014/2015/2016/2017/2018/2019/2020	MV Binary
Procedure	Humeral hemiarthroplasty/Reverse total shoulder replacement/Conventional total shoulder replacement	MV binary
Operation funding	NHS/Private	Binary
Surgical unit volume	Restricted cubic splines	Continuous
Primary surgeon grade	Consultant/Other	Binary
Consultant experience	Newly registered/Not newly registered	Binary

Table 2: Descriptive statistics of continuous variables

*Note that the mean annual consultant volume variable is a per-consultant derived variable following group mean centring. Both per-patient and per-consultant distributions of mean annual consultant volume are provided below.

Variable	Mean	SD	Q1	Median	Q3
Age	72.37	9.97	67.00	74.00	79.00
Consultant volume in the previous 365 days	22.50	13.32	12.00	21.00	31.00
Surgical unit volume in the previous 365 days	35.65	30.49	15.00	28.00	45.00
*Mean annual consultant volume (per patient)	22.51	11.41	13.52	21.32	30.20
*Mean annual consultant volume (per consultant)	12.00	10.49	3.64	9.43	17.52

Table 3: Descriptive statistics of categorical variables

Variable	Factor	N	%
Sex	Male	12089	30.78
	Female	27192	69.22
American Society of Anaesthesiologists (ASA) grade	1	2691	6.84
	2	24934	63.48
	3	11358	28.91
	4+5	298	0.76
Main surgical indication	Avascular necrosis	1013	2.58
	Cuff tear arthropathy	10695	27.23
	Inflammatory arthropathy	1534	3.91
	Osteoarthritis	22164	56.42
	Other	1067	2.72
	Trauma sequelae	2808	7.15
Previous shoulder surgery	Yes	31965	81.38
	No	7316	18.62
Surgical approach	Deltopectoral	33103	84.27
	Superior (MacKenzie)	5801	14.77

	Other	377	0.96
General anaesthetic	Yes	36058	91.8
	No	3223	8.2
Regional anaesthetic	Yes	24551	62.5
	No	14730	37.5
Chemical thromboprophylaxis	Yes	28067	71.45
	No	11214	28.55
Mechanical thromboprophylaxis	Yes	38159	97.14
	No	1122	2.86
Year of surgery	2013	2309	5.88
	2014	4392	11.18
	2015	4836	12.31
	2016	5656	14.4
	2017	5986	15.24
	2018	6245	15
	2019	6597	16.79
	2020	3260	8.3
Procedure	Humeral hemiarthroplasty	5255	13.38
	Reverse total shoulder replacement	20538	52.28
	Conventional total shoulder replacement	13488	34.34
Operation funding	NHS	35347	89.98
	Private	3934	10.02
Primary surgeon grade	Consultant	36670	93.35
	Other	2611	6.65
Consultant experience	Newly registered	6735	17.15
	Not newly registered	32546	82.85

Table 4: 365-day consultant volume for each category of confounding variable:

Variable	Category	Consultant volume in preceding 365 days					
		N	Mean	SD	Q1	Median	Q3
Age group	<55	2142	22.9	13.1	13	22	31
	55-64	4810	22.3	13.4	12	20	30
	65-74	14033	22.5	13.3	12	21	31
	75-84	15348	22.4	13.2	12	21	31
	>=85	2948	23	13.9	12	21	32
Sex	Male	12089	22.9	13.4	12	21	31
	Female	27192	22.3	13.3	12	20	31
American Society of Anaesthesiologists (ASA) grade	1	2691	22.5	13.6	12	20	31
	2	24934	22.6	13.4	12	21	31
	3	11358	22.4	13.1	12	20	31
	4+5	298	22.2	12.1	13	21.5	30
Main surgical indication	Avascular necrosis	1013	20.2	12.2	11	18	27
	Cuff tear arthropathy	10695	23.8	13.5	13	22	33
	Inflammatory arthropathy	1534	22.2	12.5	13	21	30
	Osteoarthritis	22164	22	13.4	12	20	30
	Other	1067	24.1	13.6	13	22	34
	Trauma sequelae	2808	21.8	12.7	12	20	30
Previous shoulder surgery	Yes	7316	22.6	13.2	12	21	31
	No	31965	22.5	13.4	12	21	31
Surgical approach	Deltopectoral	33103	22	13	12	20	30

	Superior (MacKenzie)	5801	25.1	14.5	14	23	35
	Other	377	22.2	15.7	9	17	34
General anaesthetic	Yes	36058	22.5	13.4	12	20.5	31
	No	33223	22.6	12.3	13	22	31
Regional anaesthetic	Yes	24551	23	13.2	13	21	31
	No	14730	25.6	13.4	11	19	30
Chemical thromboprophylaxis	Yes	28067	22.7	13.3	12	21	32
	No	11214	21.9	13.4	12	20	29
Mechanical thromboprophylaxis	Yes	38159	22.5	13.3	12	21	31
	No	1122	21.2	12.7	11	20	30
Year of surgery	2013	2309	18.8	11.1	11	17	25
	2014	4392	20.3	11.7	11	19	28
	2015	4836	21.4	12.9	11	20	30
	2016	5656	22.3	13.5	12	20	30
	2017	5986	23.8	14.2	13	21	33
	2018	6245	23.5	13.2	13	22	32
	2019	6597	25	13.8	14	23	34
	2020	3260	20.8	13.4	11	19	27
Procedure	Humeral hemiarthroplasty	5255	16.9	11.6	8	15	23
	Reverse total shoulder replacement	20538	23.9	13.7	13	22	33
	Conventional total shoulder replacement	13488	22.5	12.7	13	21	31
Operation funding	NHS	35347	22.6	13.4	12	21	31
	Private	3934	21.7	13	12	20	30

Surgical unit volume (preceding 365 days)	<25	17038	17.4	11.5	9	15	24
	25-49	13702	23.4	12.3	14	22	31
	50-99	6514	29.5	13.4	20	28	38
	>=100	2027	36.4	12	29	36	44
Primary surgeon grade	Consultant	36670	22.3	13.3	12	20	31
	Other	2611	25.2	13.2	15	24	34
Consultant experience	Newly registered	6735	18.3	12.4	9	16	26
	Not newly registered	32546	23.4	13.3	13	22	32

Table 5: Descriptive statistics by mean annual consultant volume, categorised by 25th, 50th and 75th percentiles- patient age at primary:

Note that the mean annual consultant volume variable is a per-consultant derived variable following group mean centring. Patient-level descriptive statistics are reported in the table below, meaning the distribution of the mean annual consultant volume appears different to that in Figure 3 in the main manuscript.

**Mean annual consultant volume categories**	**Patient age at primary**				
	**Mean**	**SD**	**Q1**	**Median**	**Q3**
**<=13.5** (n=9870)	72.37	9.8	67	74	79
**13.5-21.3** (n=9857)	72.44	9.98	67	74	79
**21.3-30.2** (n=9854)	72.21	10.16	67	74	79
**>=30.2** (n=9700)	72.44	9.95	67	74	79

Table 6: Descriptive statistics by mean annual consultant volume, categorised by 25th, 50th and 75th percentiles - categorical variables:

Note that the mean annual consultant volume variable is a per-consultant derived variable following group mean centring. Patient-level descriptive statistics are reported in the table below, meaning the distribution of the mean annual consultant volume appears different to that in Figure 3 in the main manuscript.

Variable	Factor	Mean annual consultant volume categories							
		<=13.5 (n=9870)		13.5-21.3 (n=9857)		21.3-30.2 (n=9854)		>=30.2 (n=9700)	
		N	%	N	%	N	%	N	%
Sex	Male	2885	29.23	3044	30.88	3045	30.9	3115	32.11
	Female	6985	70.77	6813	69.12	6809	69.1	6585	67.89
American Society of Anaesthesiologists (ASA) grade	1	646	6.55	708	7.18	628	6.37	709	7.31
	2	6249	63.31	6123	62.12	6291	63.84	6271	64.65
	3	2904	29.42	2945	29.88	2850	28.92	2659	27.41
	4+5	71	0.72	81	0.82	85	0.86	61	0.63
Main surgical indication	Avascular necrosis	323	3.27	278	2.82	226	2.29	186	1.92
	Cuff tear arthropathy	2319	23.5	2462	24.98	2976	30.2	2938	30.29
	Inflammatory arthropathy	354	3.59	401	4.07	455	4.62	324	3.34
	Osteoarthritis	5868	59.45	5721	58.04	5191	52.68	5384	55.51
	Other	254	2.57	240	2.43	312	3.17	261	2.69
	Trauma sequelae	752	7.62	755	7.66	694	7.04	607	6.26
Previous shoulder surgery	Yes	1825	18.49	1704	17.29	1947	19.76	1840	18.97
	No	8045	81.51	8153	82.71	7907	80.24	7860	81.03
Surgical approach	Deltopectoral	8658	87.72	8528	86.52	8148	82.69	7769	80.09
	Superior (MacKenzie)	1095	11.09	1251	12.69	1614	16.38	1841	18.98
	Other	117	1.19	78	0.79	92	0.93	90	0.93
General anaesthetic	Yes	9167	92.88	9243	93.77	8600	87.27	9048	93.28
	No	703	7.12	614	6.23	1254	12.73	652	6.72
Regional anaesthetic	Yes	5553	56.26	6165	62.54	6634	67.32	6199	63.91
	No	4317	43.74	3692	37.46	3220	32.68	3501	36.09

Chemical thromboprophylaxis	Yes	7234	73.29	6101	61.9	7288	73.96	7444	76.74
	No	2636	26.71	3756	38.1	2566	26.04	2256	23.26
Mechanical thromboprophylaxis	Yes	9555	96.81	9538	96.76	9588	97.3	9478	97.71
	No	315	3.19	319	3.24	266	2.7	222	2.29
Year of surgery	2013	474	4.8	666	6.76	541	5.49	628	6.47
	2014	1024	10.37	1172	11.89	1098	11.14	1098	11.32
	2015	1172	11.87	1159	11.76	1219	12.37	1286	13.26
	2016	1410	14.29	1381	14.01	1405	14.26	1460	15.05
	2017	1513	15.33	1510	15.32	1506	15.28	1457	15.02
	2018	1586	16.07	1528	15.5	1659	16.84	1472	15.18
	2019	1783	18.06	1623	16.47	1672	16.97	1519	15.66
	2020	908	9.2	818	8.3	754	7.65	780	8.04
Procedure	Humeral hemiarthroplasty	2133	21.61	1583	16.06	772	7.83	767	7.91
	Reverse total shoulder replacement	4620	46.81	4809	48.79	5612	56.95	5497	56.67
	Conventional total shoulder replacement	3117	31.58	3465	35.15	3470	35.21	3436	35.42
Operation funding	NHS	8873	89.9	8815	89.43	8982	91.15	8677	89.45
	Private	997	10.1	1042	10.57	872	8.85	1023	10.55
Primary surgeon grade	Consultant	9389	95.13	9327	94.62	9161	92.97	8793	90.65
	Other	481	4.87	530	5.38	693	7.03	907	9.35
Consultant experience	Newly registered	2772	28.09	1486	15.08	1368	13.88	1109	11.43
	Not newly registered	7098	71.91	8371	84.92	8486	86.12	8591	88.57

Table 7: Group mean centring:

Worked example of group mean centring. The mean annual consultant volume and deviation annual consultant volume are generated for each procedure undertaken by one hypothetical consultant surgeon over their career.

Operation date	Primary exposure: surgical volume in previous 365 days	Mean annual consultant volume	Deviation annual consultant volume
01/03/2013	*(<365 days of data)*		
05/03/2013	*(<365 days of data)*		
27/02/2014	2	1.86	+0.14
05/08/2014	1	1.86	-0.86
01/09/2015	0	1.86	-1.86
10/10/2015	1	1.86	-0.86
12/10/2015	2	1.86	+0.14
13/12/2015	3	1.86	+1.14
30/12/2015	4	1.86	+2.14

Table 8: OPCS-4 codes to identify reoperations

Procedure type	OPCS-4 codes	Anatomy codes
SAD/ACJ excision	O291	
	W572,W844	Z812,Z814,Z891
	T621,T622,T626,T628,T629	Z814,Z891
Rotator cuff repair	T791,T793,T794,T795	
	T641,T642,T643,T744,T648,T649,T67, T68	Z742
MUA +-release	W911,W913,W918,W919,W781,W784	Z813, Z814, Z891
	W911,W913, W918, W919, W781, W784	Z813, Z814, Z891
Washout/debridement	W80, W811, W812, W813, W815, W713	Z813, Z814, Z891
	Y223, Y311, Y318, Y319, Y321	Z813, Z814, Z891
	W18	Z691, Z692, Z693, Z694
Synovectomy	W691, W692, W693, T711	Z813, Z814, Z891
Osteomyelitis surgery	W18	Z691, Z692, Z693,Z694
Complex reconstruction	O108, O109	
Bone resection	W068, W069, W091, W092, W093, W094	Z691, Z692, Z693
	W095, W096, W097, W098, W099	Z691, Z692, Z693
Arthroscopy or other soft tissue	Y528,Y767	Z812, Z813, Z814, Z891
	W816,W817,W818,W819	Z812, Z813, Z814, Z891
	W83	Z812, Z813, Z814, Z891
	W843,W845,W846W,847W,848W,868 W,869	Z812, Z813, Z814, Z891
	W881,W888,W889	Z812, Z813, Z814, Z891

	W891,W898,W899,O198,O199	Z812, Z813, Z814, Z891
	W714,W718,W719	Z812, Z813, Z814, Z891
	W694,W695,W698,W699,W711,W712	Z812, Z813, Z814, Z891
	T645,T651,T658,T659,T701,T702	Z812, Z813, Z814, Z891
	W562,W563,W564,W568,W569	Z812, Z813, Z814, Z891
Surgery for instability	O27	
	W77 (Not W776), W841, W842	
	W72, W73, W74, W75	Z813, Z814, Z891
Reduction of dislocation	W652, W658, W689, W662, W668	Z813, Z814, Z891
	W669, W672, W674, W678, W679	Z813, Z814, Z891
Fixation of periprosthetic fracture	W19 (not W191), W20, W21, W22	Z691, Z692, Z693, Z813, Z814, Z891
	W23, W24 (not W241), W25, W26	Z691, Z692, Z693, Z813, Z814, Z891
	W651, W653, W654, W656, W661, W663, W664	Z691, Z692, Z693, Z813, Z814, Z891
	W671, W673, W677, W332	Z691, Z692, Z693, Z813, Z814, Z891
	O172, O173, O175, O178, O179	Z691, Z692, Z693, Z813, Z814, Z891

Table 9: ICD-10 codes to identify serious adverse events

**Event**	**ICD-10 codes**
Pulmonary elbolism	I26
Myocardial infarction	I21,I22
Cerebrovascular event	I60,I61,I62,I63,I64
Acute kidney injury	N17
Lower respiratory tract infection	J12,J13,J14,J15,J16,J18,J22,J86,J440,J851,J690
Urinary tract infection	N10,N300,N308,N309,N390
Death	*Civil Registration Mortality data linked to NJR data*

Multilevel models

Primary outcome (Revision): Multilevel (Weibull) survival model

Denote by V_{ij} the volume in the 365 days prior to procedure i (i = 1, … , I) carried out by surgeon j (j = 1, … , J) and $\bar{V}_j$ the surgeon's mean annual volume over the observation period. A multilevel Weibull
model for the association between $h_{ij}(t)$, the hazard of revision at time t for procedure i of surgeon j,
and volume is specified as

$$h_{ij}(t) = h_0(t)\exp\left[\alpha S(\bar{V}_j, K_0) + \gamma S\left((\bar{V}_j - V_{ij}), K_1\right) + \beta X_{ij} + Z_j\right]$$

where $h_0(t)$ is the baseline hazard function of a Weibull distribution $S()$ represents the restricted cubic spline basis, with a vector of Kk knots, α is a vector of coefficients representing the mean annual consultant volume association, γ is a vector of coefficients for the deviation annual consultant volume association, and β is a vector of coefficients for confounding factors X_{ij} , and Z_j ~ $N(0, \sigma^2)$ is a surgeon-level random effect.

Secondary outcomes (reoperations, serious adverse events, prolonged hospital stay): Multilevel logistic model

Denote by V_{ij} the volume in the 365 days prior to procedure i (i = 1, … , I) carried out by surgeon j (j = 1, … , J) and $\bar{V}_j$ the surgeon's mean annual volume over the observation period. A multilevel logistic
model for the association between the log odds of the secondary outcome event where p_{ij} is the probability of the event for procedure *i* of surgeon *j*, and volume is specified as

$$\log\left(\frac{p_{ij}}{1 - p_{ij}}\right) = \alpha S(\bar{V}_j, K_0) + \gamma S\left((\bar{V}_j - V_{ij}), K_1\right) + \beta X_{ij} + Z_j$$

where $S()$ represents the restricted cubic spline basis, with a vector of Kk knots, α is a vector of coefficients representing the mean annual consultant volume association, γ is a vector of coefficients for the deviation annual consultant volume association, and β is a vector of coefficients for confounding factors and the intercept X_{ij} and Z_j ~ $N(0, \sigma^2)$ is a surgeon-level random effect.

Incremental confounding adjustment for primary and secondary outcomes

Note:

Adjustment-0 was the crude model

Adjustment-1 included confounding adjustment for patient factors

Adjustment-2 included confounding adjustment for patient factors, operation factors

Adjustment-3 included confounding adjustment for patient factors, operation factors, centre factors

Adjustment-4 included confounding adjustment for patient factors, operation factors, centre factors, consultant factors

Revision:

Mean annual consultant volume confounding adjustment (threshold: 10.68, 11.13, 9.83, 10.85, 10.41):

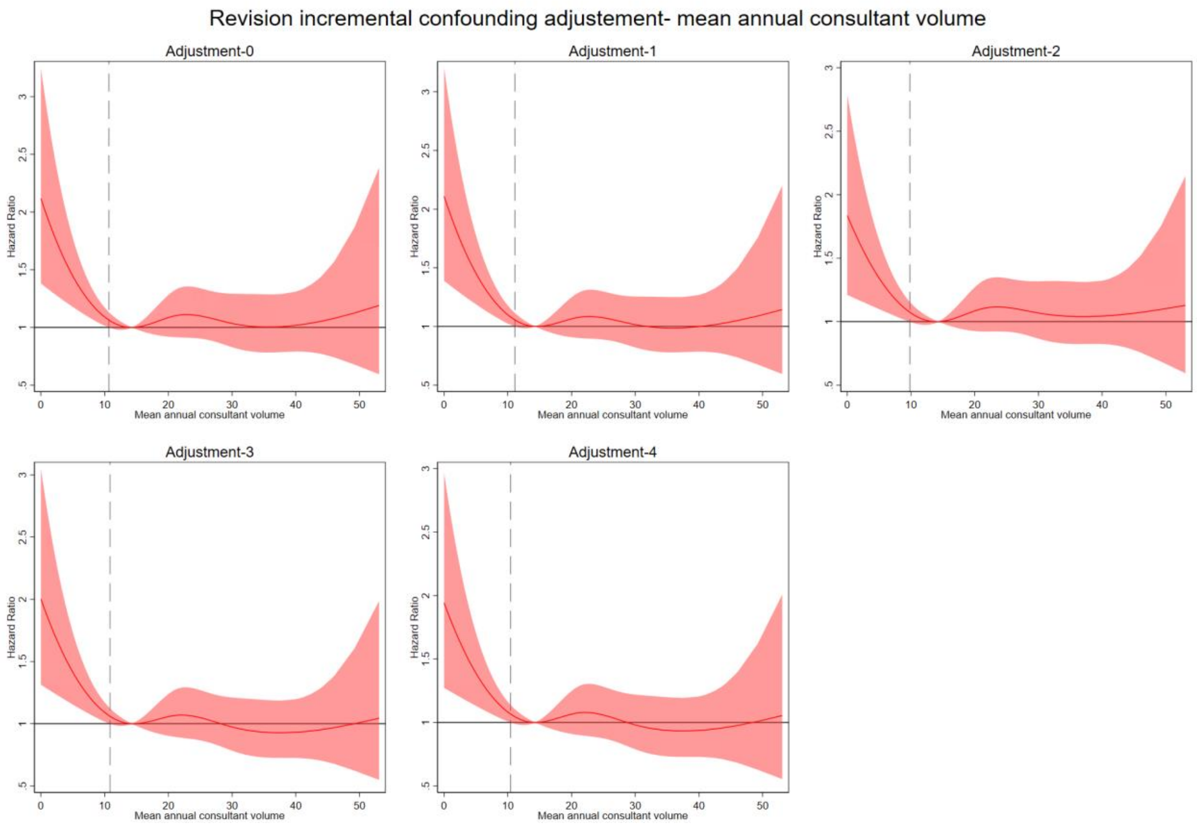

Deviation annual consultant volume confounding adjustment:

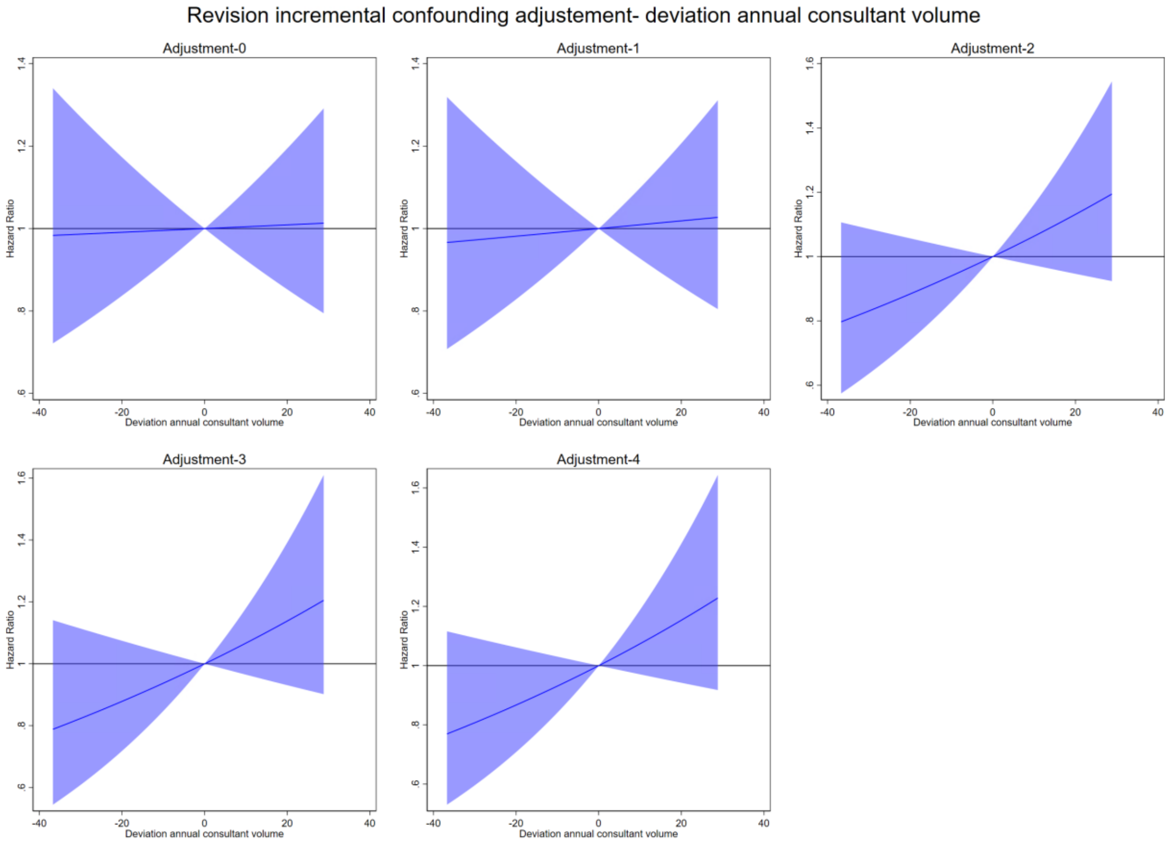

30-day serious adverse events (SAE):

Mean annual consultant volume confounding adjustment:

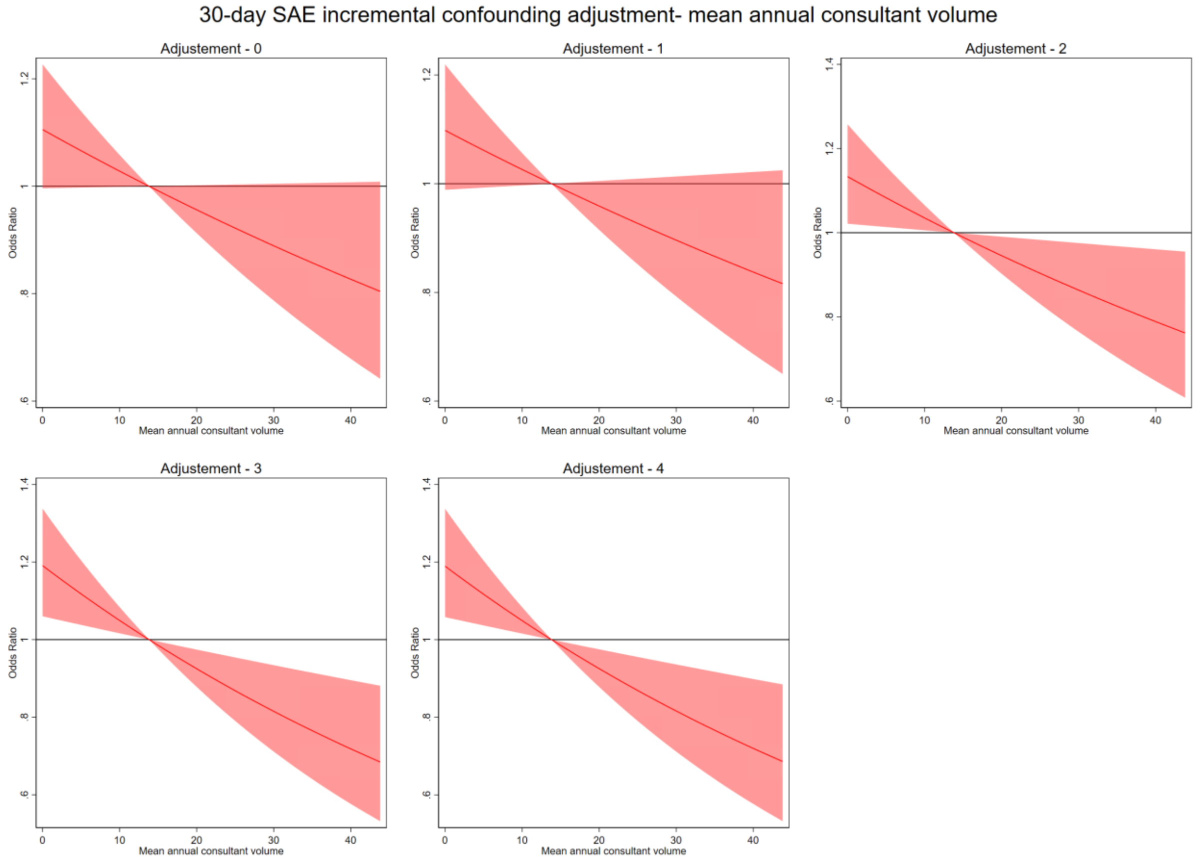

Deviation annual consultant volume confounding adjustment:

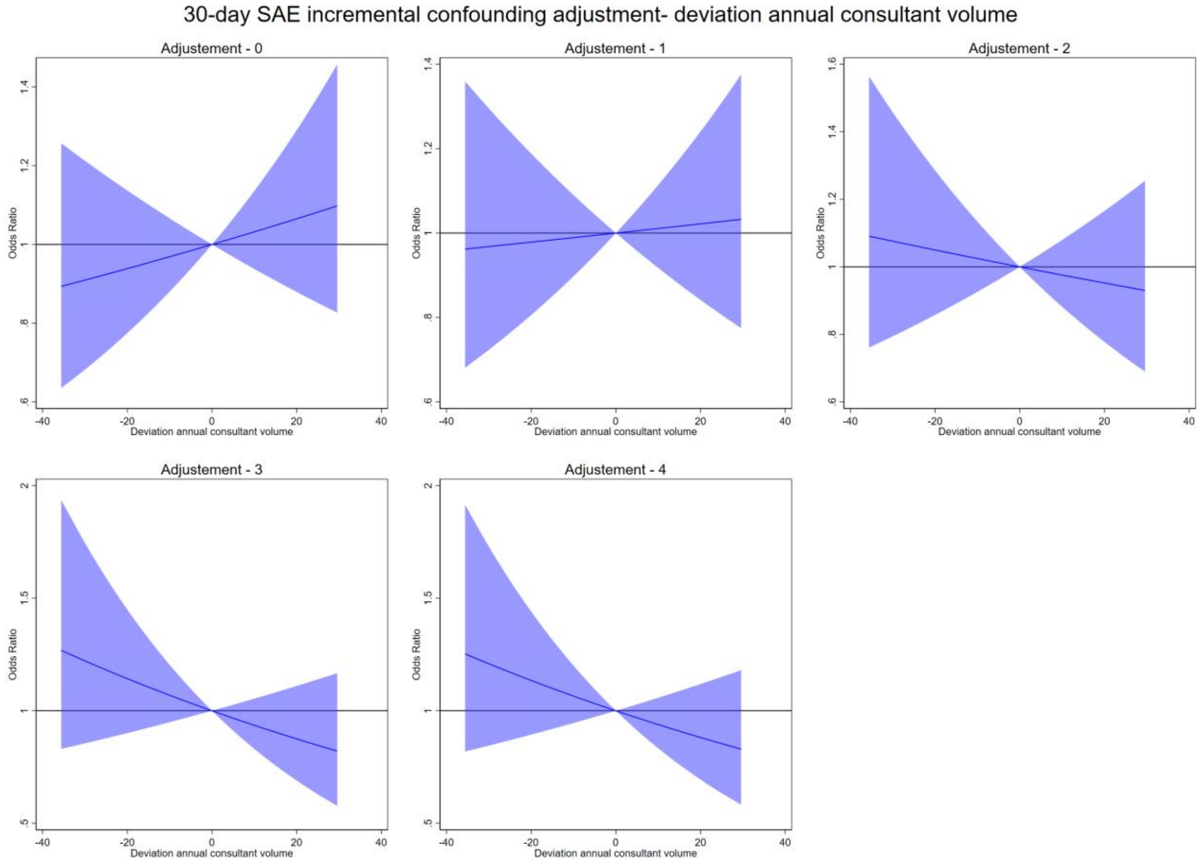

90-day serious adverse events (SAE):

Mean annual consultant volume confounding adjustment:

90-day SAE incremental confounding adjustment- mean annual consultant volume

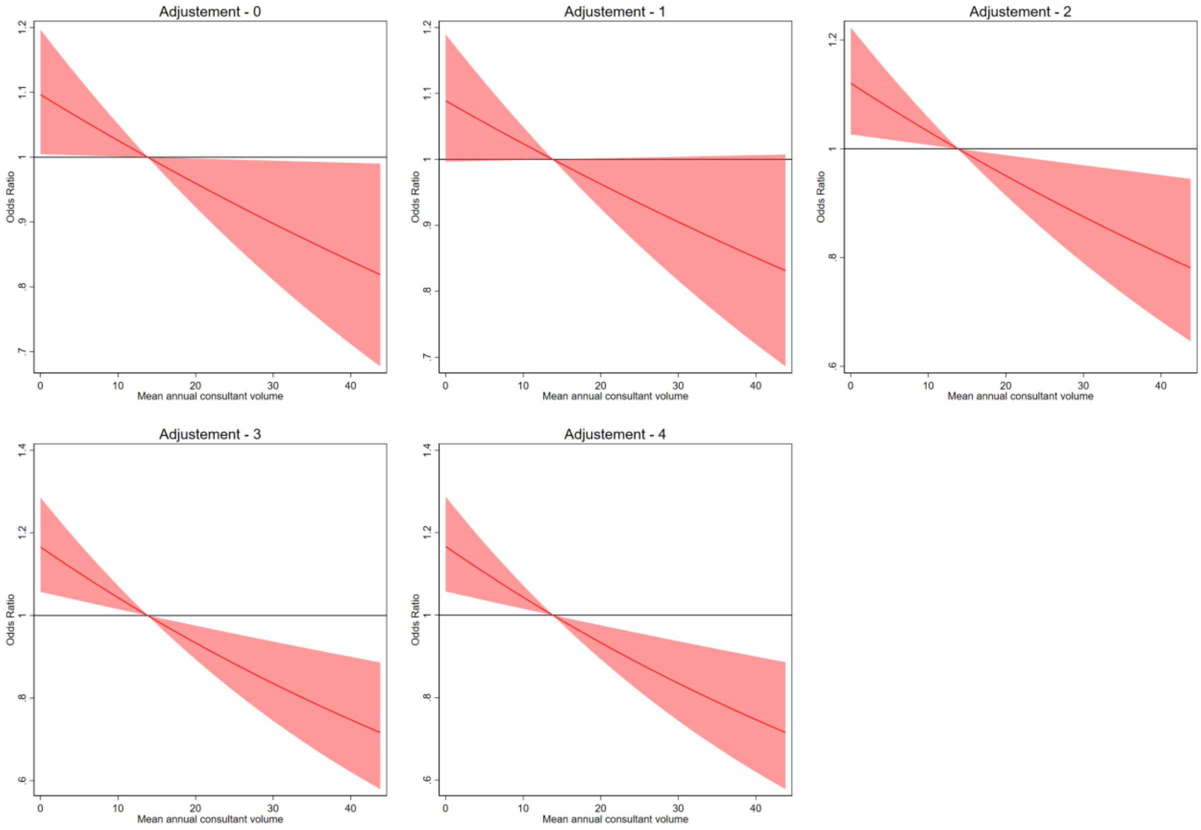

Deviation annual consultant volume confounding adjustment:

90-day SAE incremental confounding adjustment- deviation annual consultant volume

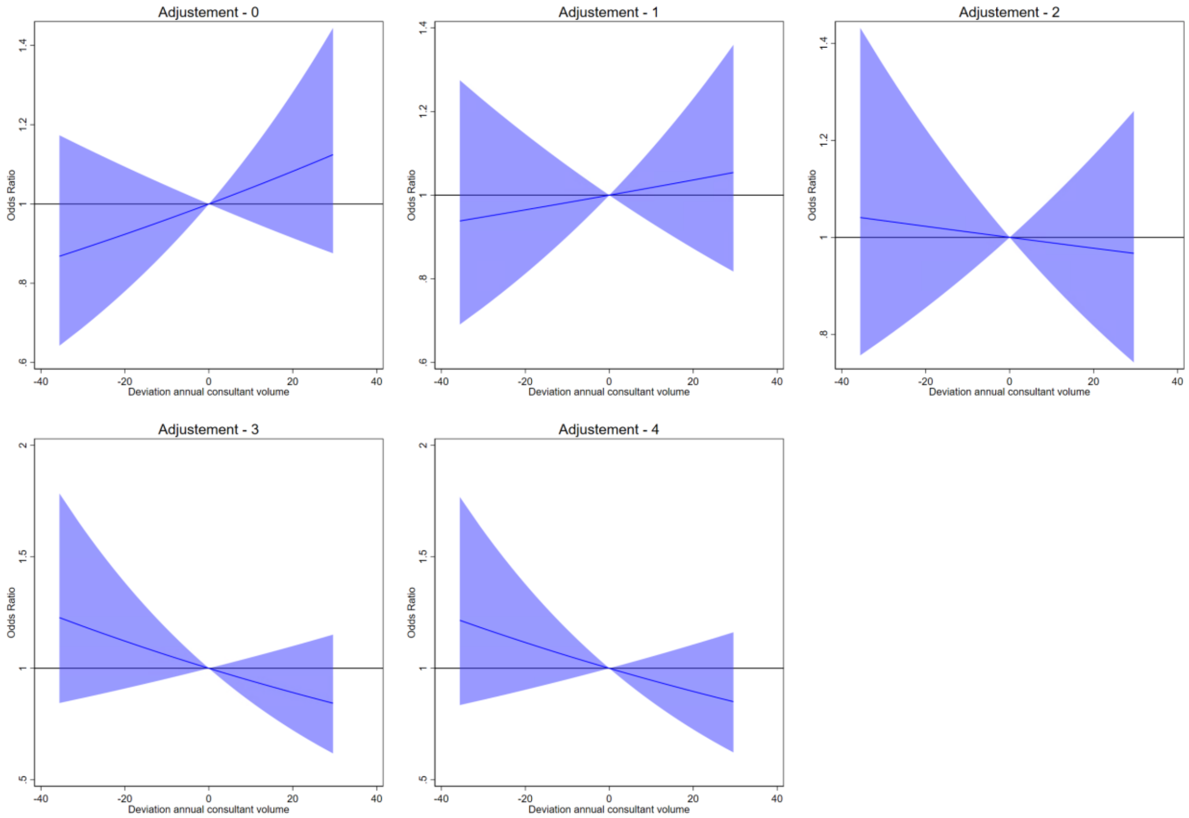

Prolonged hospital stay (>3 nights):

Mean annual consultant volume confounding adjustment:

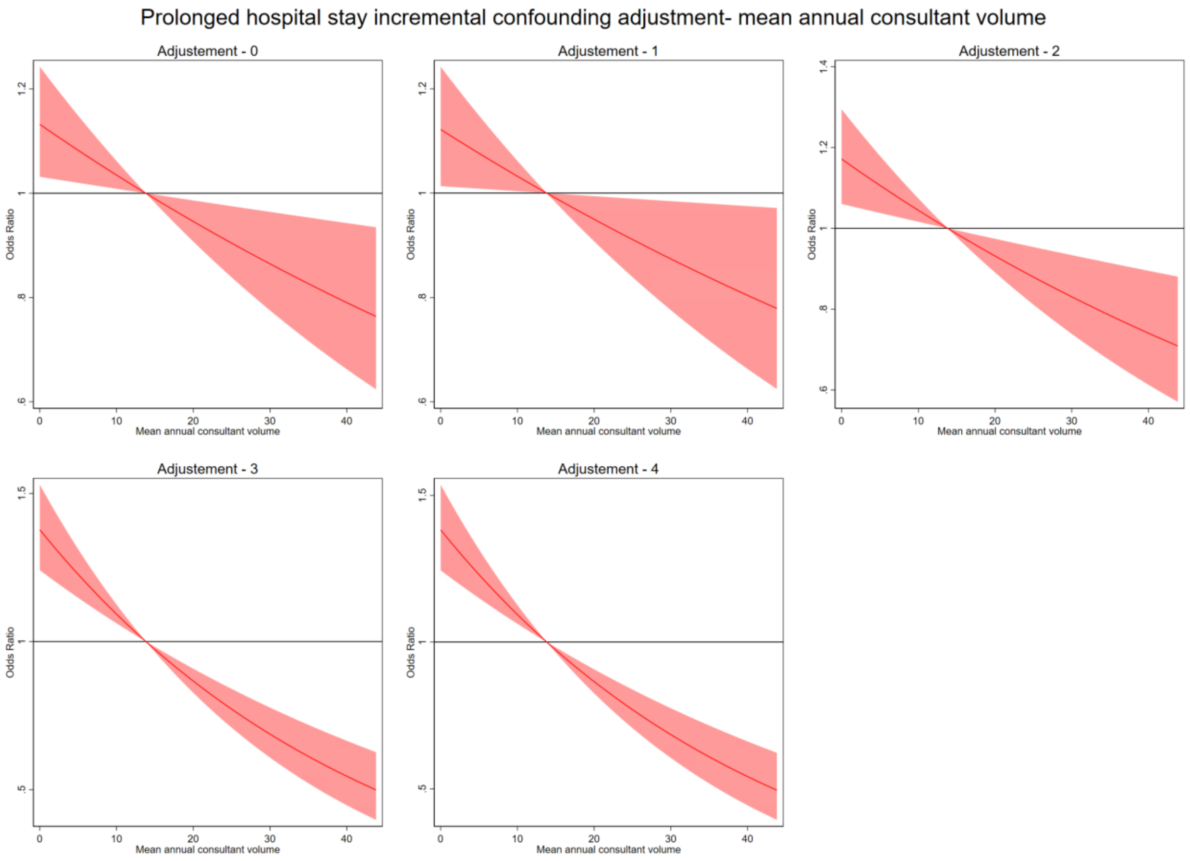

Deviation annual consultant volume confounding adjustment:

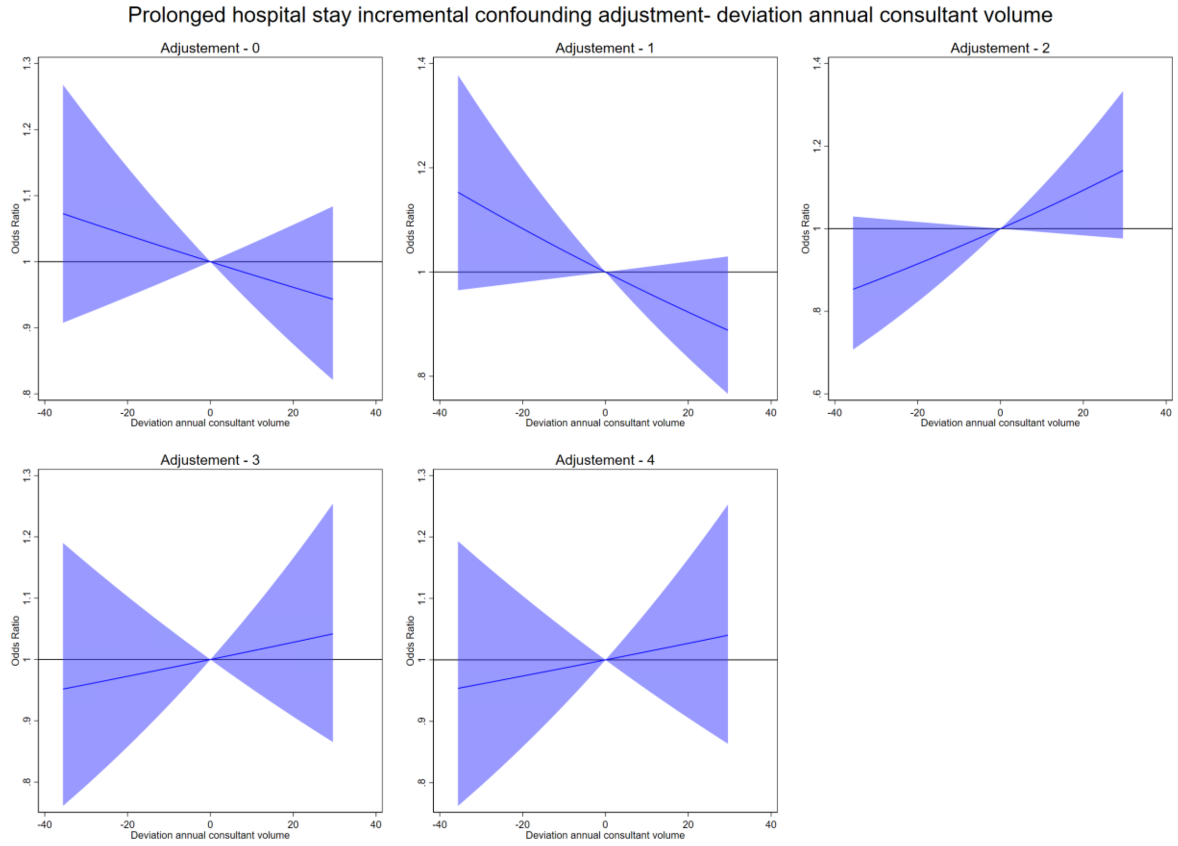

Reoperations:

Mean annual consultant volume confounding adjustment:

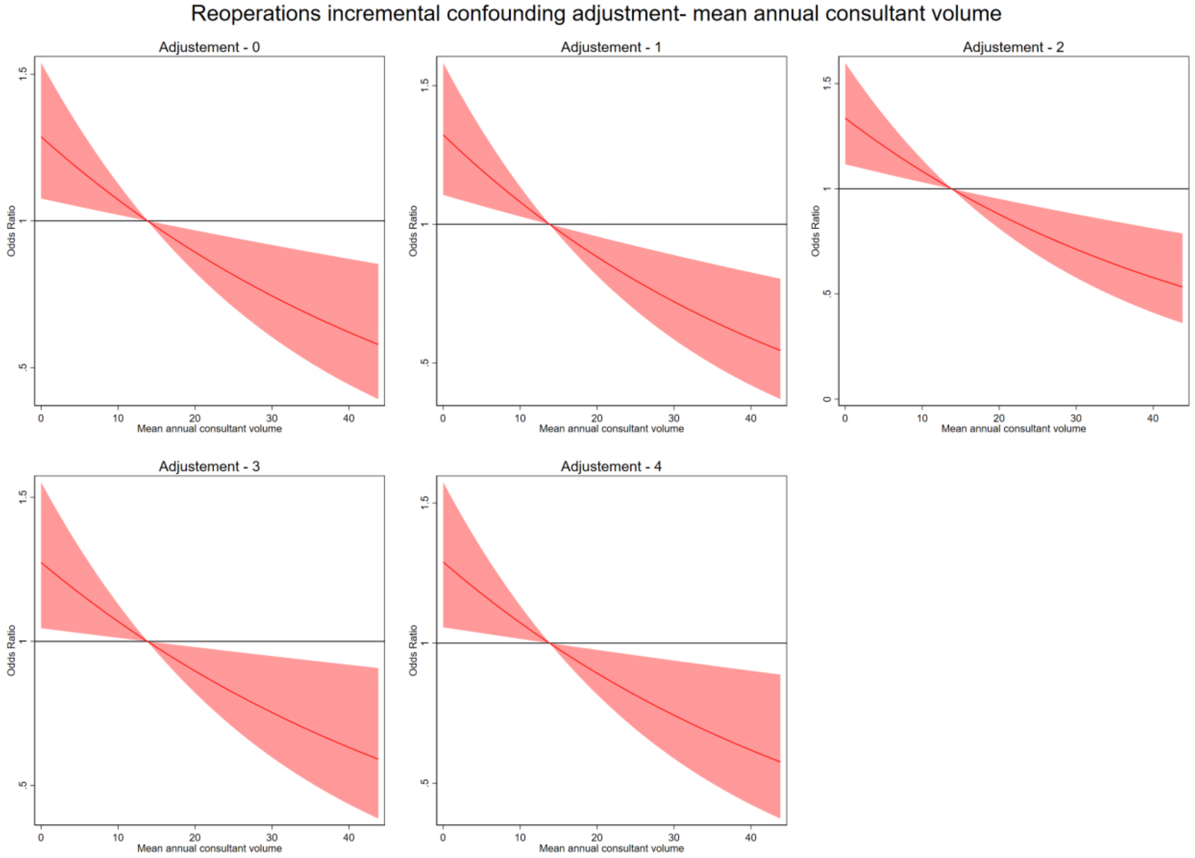

Deviation annual consultant volume confounding adjustment:

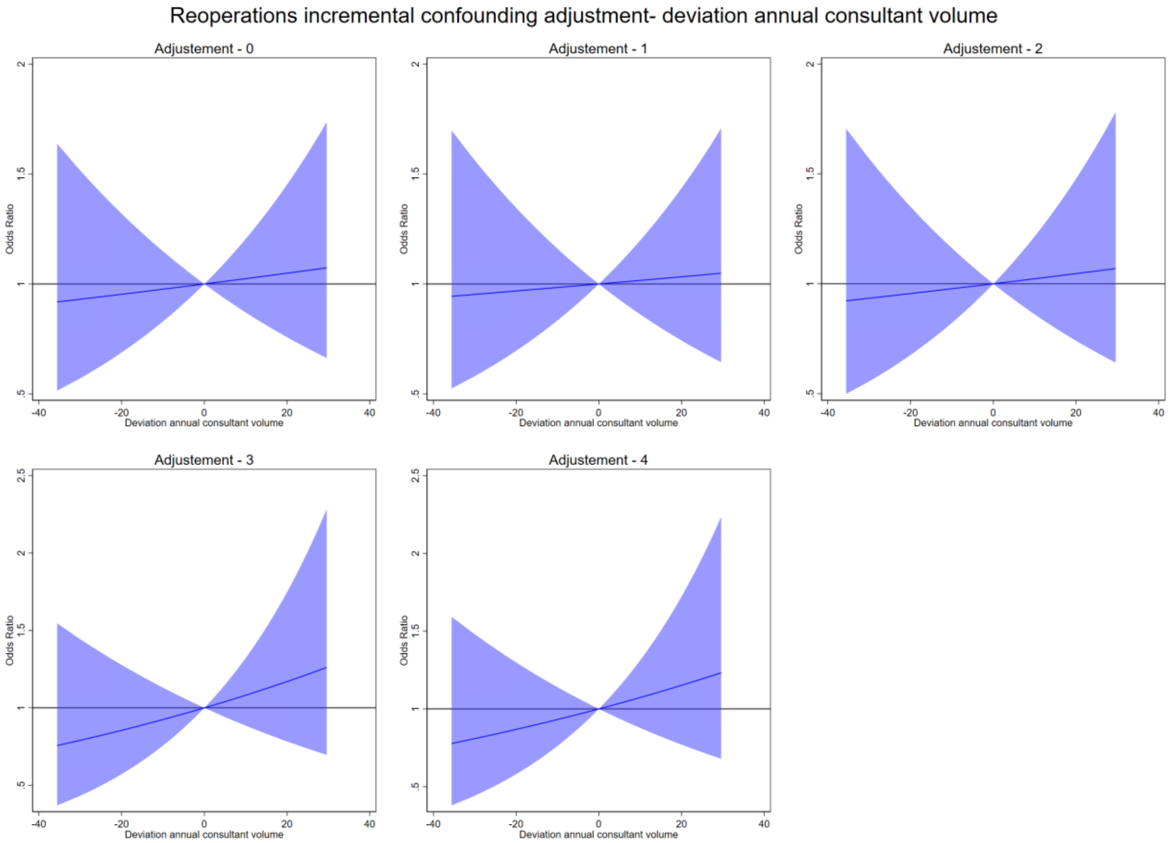

Sensitivity analysis

Additional adjustment for the following covariates was undertaken for the secondary outcomes using the linked NJR/HES dataset:

1. Ethnic group (categorical variable: Asian or Asian British; Black, Black British, Caribbean or African; Mixed or multiple ethnic groups; White; Other ethnic group)

2. Region of treatment (categorical variable: Eastern; London; North West; Northern and Yorkshire; South East; South West; Trent; West Midlands)

3. Index of multiple deprivation (IMD) (ordinal variable: calculated by ranking the 32,844 lower layer super output areas (LSOA) in England from most deprived to least deprived and dividing them into 10 equal groups)

Ethnic group was missing in 2,900 cases (9.2%) and IMD was missing in 416 cases (1.3%).

These three additional covariates were adjusted for by adding them to the fully adjusted models (that already included patient factors, operation factors, centre factors, consultant factors). Each of these additional covariates was adjusted for separately, and then all were adjusted for together, to demonstrate their effects on the secondary outcomes. Five analyses were undertaken for the sensitivity analysis:

1. Complete case analysis: adjusting for IMD only (n=30,991)
2. Complete case analysis: adjusting for ethnic group only (n=28,507)
3. Complete case analysis: adjusting for treatment region only (n=31,407)
4. Multiple imputation of ethnic group and IMD: adjusting for ethnic group only (n=31,407)
5. Multiple imputation of ethnic group and IMD: adjusting for all three additional covariates together (n=31,407)

The relative risk reduction from a mean consultant volume of 0 to 40 procedures per year is reported in each analysis in the tables accompanying the graphs below.

Complete case analysis: adjusting for IMD only (n=30,991)

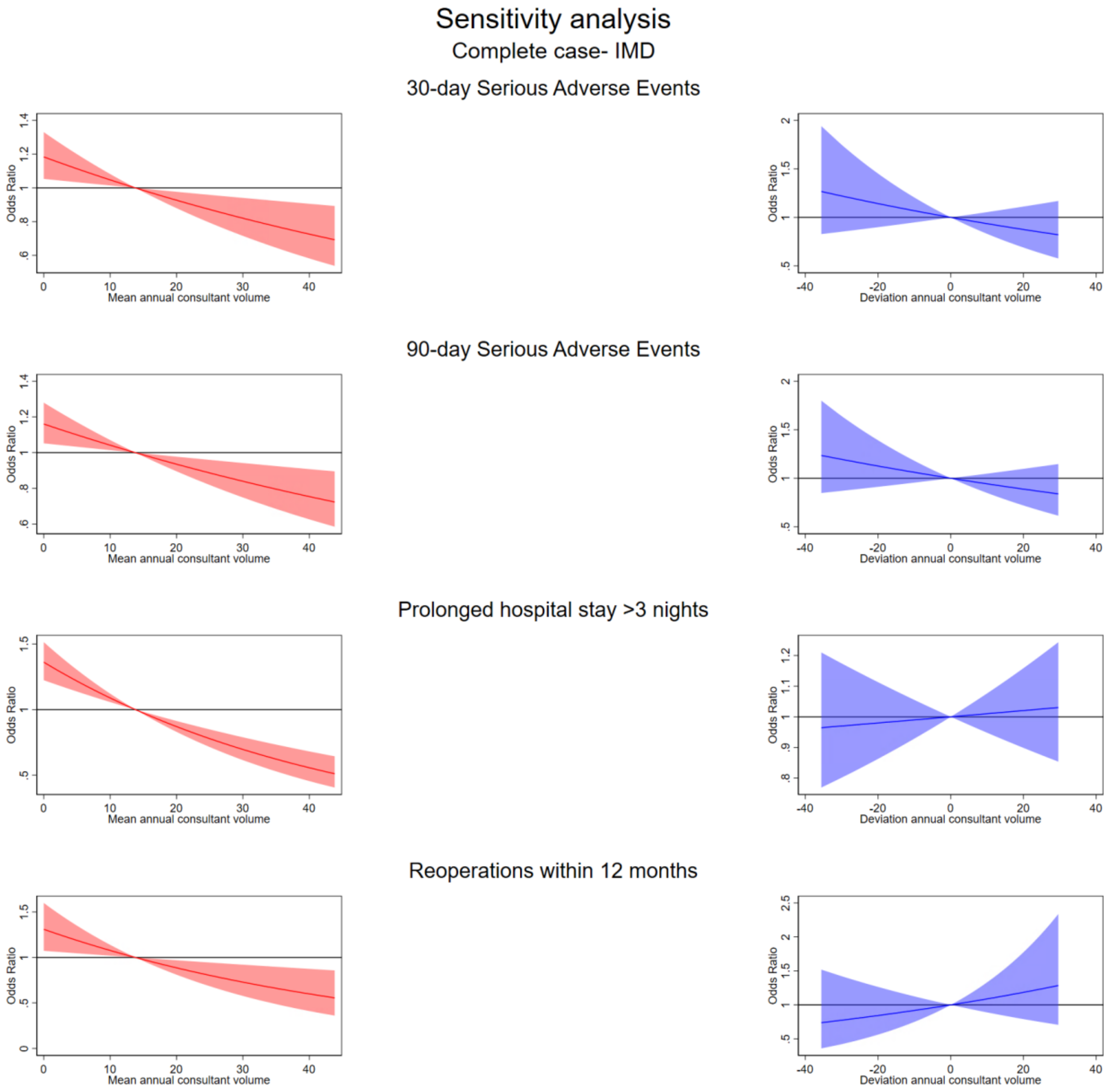

Complete case: adjusting for IMD only (n=30,991)			
	Odds risk: mean annual consultant volume		% risk reduction
	0 procedures /year	40 procedures /year	
30-day Serious Adverse Events	1.18	0.72	39.6
90-day Serious Adverse Events	1.16	0.74	35.9
Prolonged hospital stay	1.36	0.54	60.1
Reoperation within 12 months	1.31	0.59	55.3

Complete case analysis: adjusting for ethnic group only (n=28,507)

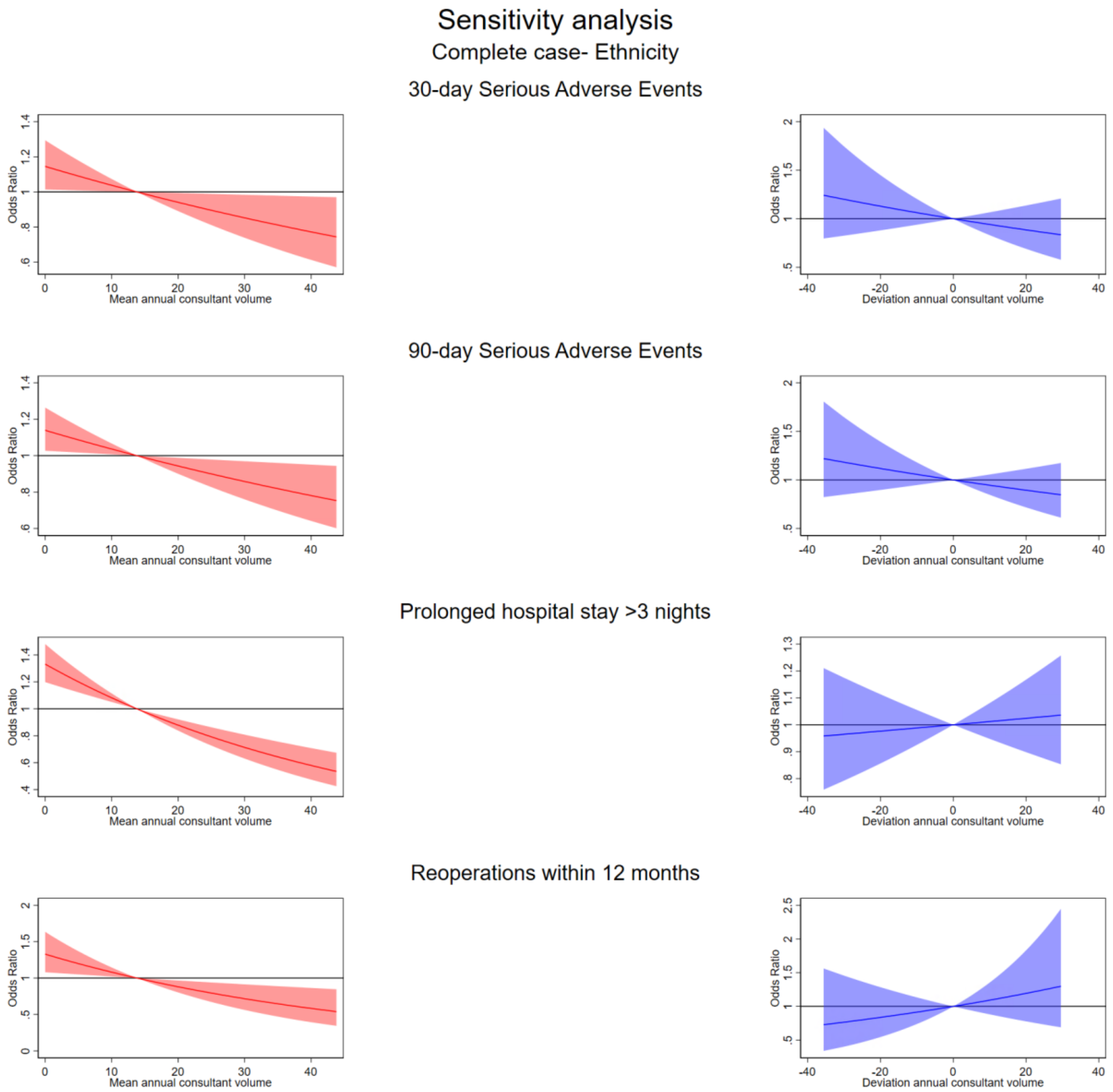

Complete case: adjusting for ethnic group only (n=28,507)			
	Odds risk: mean annual consultant volume		% risk reduction
	0 procedures /year	40 procedures /year	
30-day Serious Adverse Events	1.15	0.76	33.3
90-day Serious Adverse Events	1.14	0.77	32.2
Prolonged hospital stay	1.33	0.57	57.5
Reoperation within 12 months	1.33	0.57	57.1

Complete case analysis: adjusting for treatment region only (n=31,407)

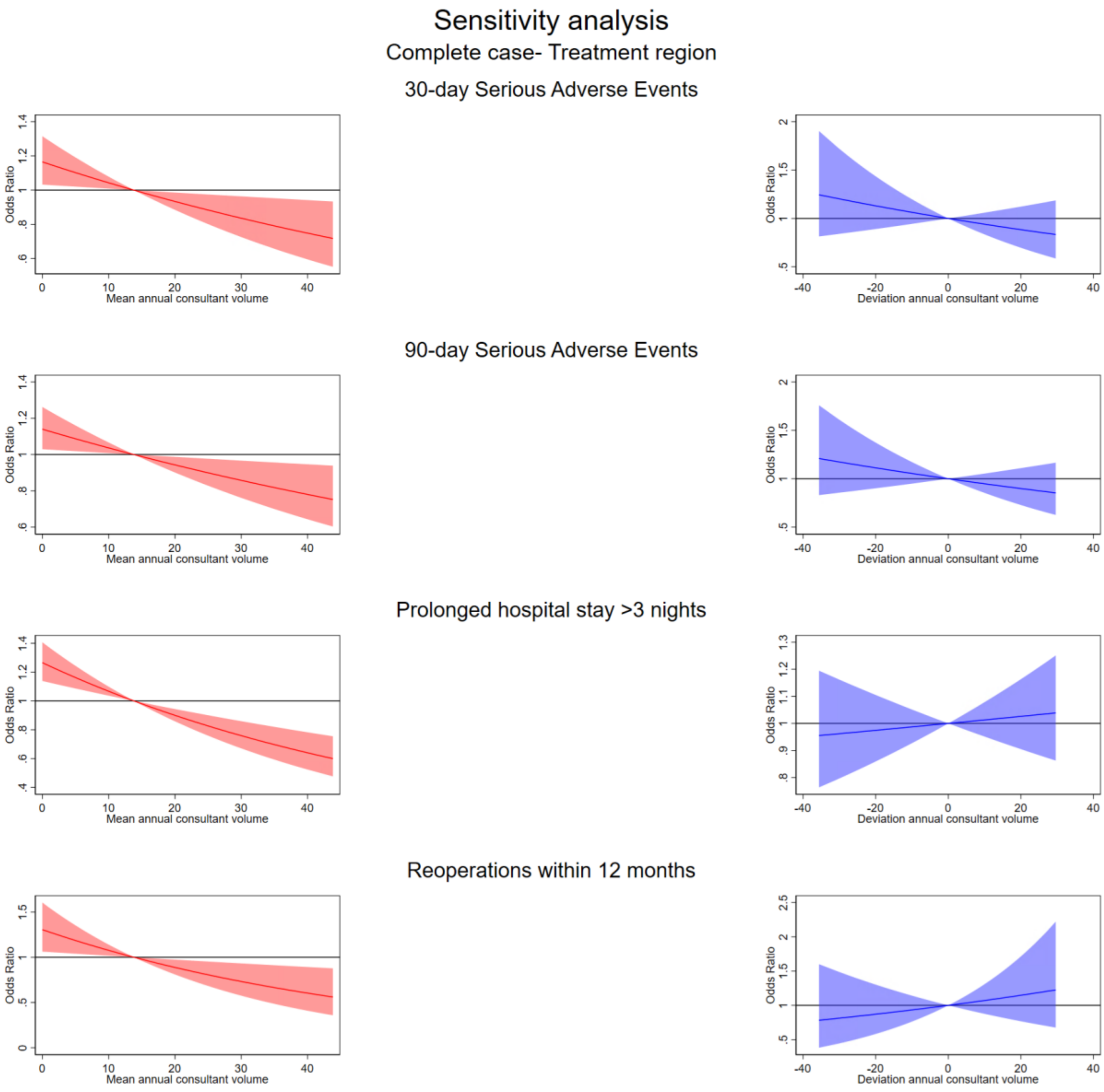

Complete case: adjusting for treatment region only (n=31,407)			
	Odds risk: mean annual consultant volume		% risk reduction
	0 procedures /year	40 procedures /year	
30-day Serious Adverse Events	1.17	0.74	36.6
90-day Serious Adverse Events	1.14	0.77	32.3
Prolonged hospital stay	1.27	0.63	50.4
Reoperation within 12 months	1.31	0.59	54.8

Multiple imputation of ethnic group and IMD: adjusting for ethnic group only (n=31,407)

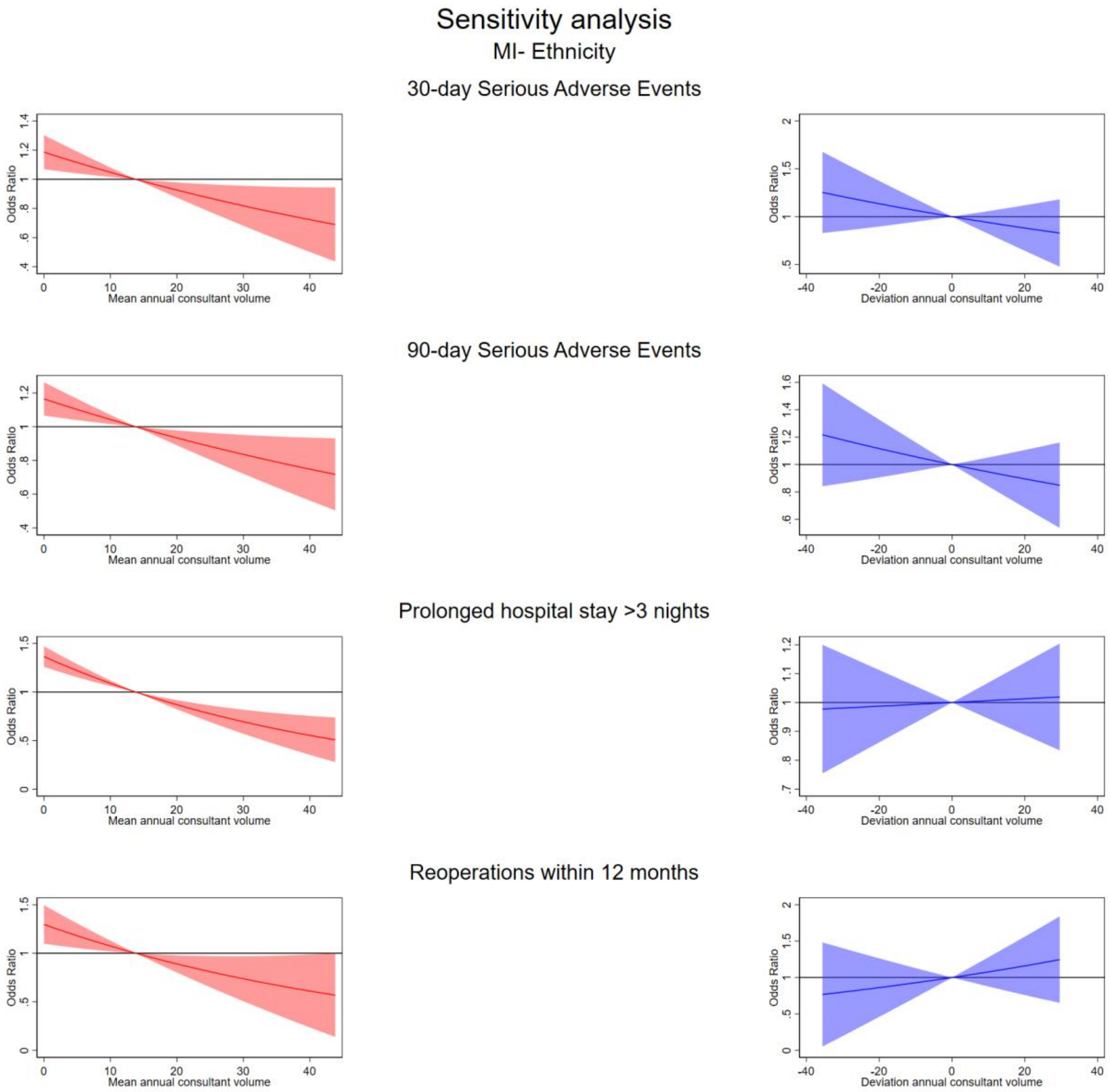

Multiple imputation: adjusting for ethnic group only (n=31,407)			
	Odds risk: mean annual consultant volume		
	0 procedures /year	40 procedures /year	% risk reduction
30-day Serious Adverse Events	1.19	0.71	40.0
90-day Serious Adverse Events	1.17	0.74	36.6
Prolonged hospital stay	1.37	0.54	60.5
Reoperation within 12 months	1.30	0.60	53.9

Multiple imputation of ethnic group and IMD: adjusting for all three additional covariates together (n=31,407)

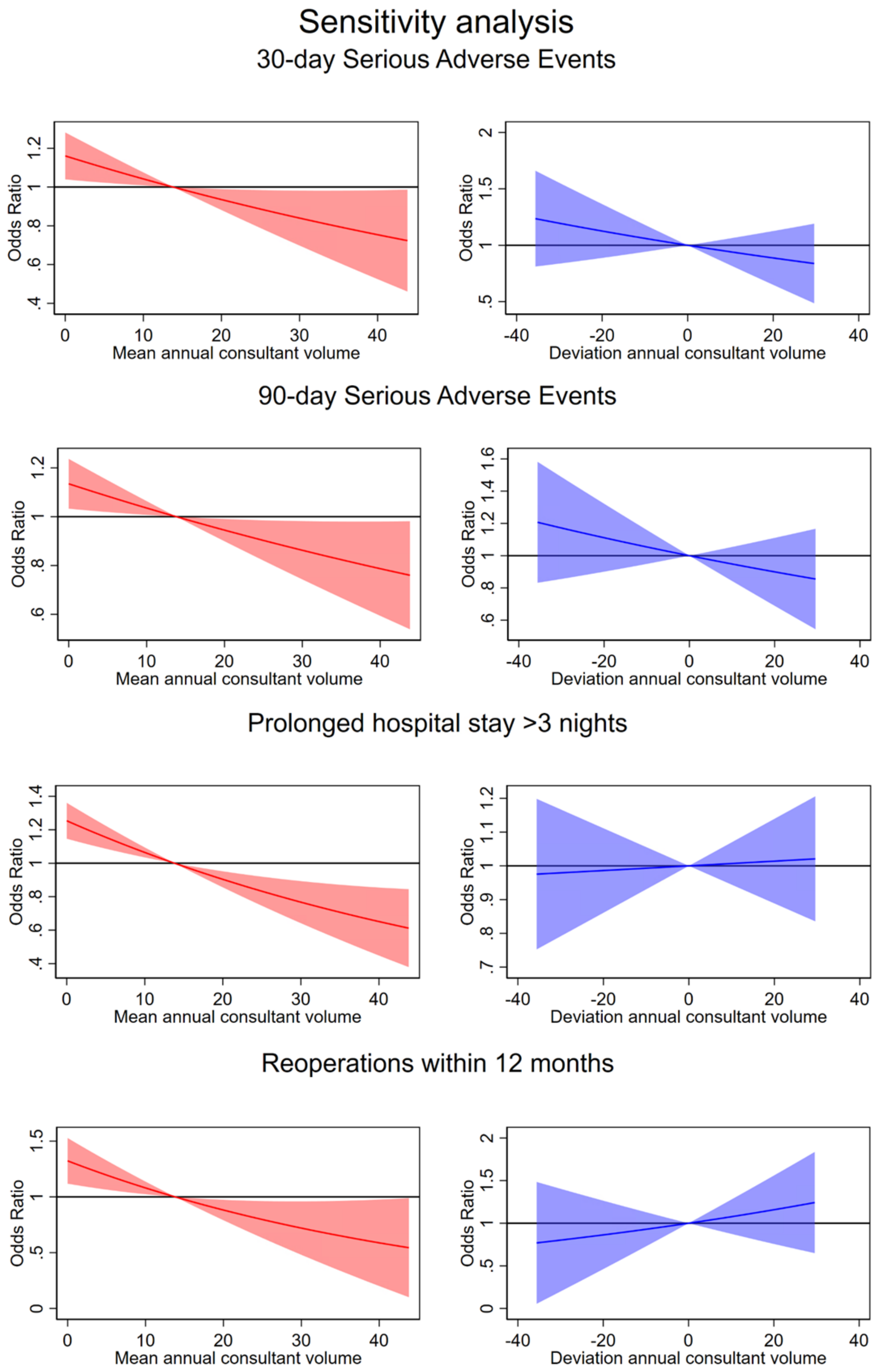

Multiple imputation: adjusting for all three additional covariates together (n=31,407)			
	Odds risk: mean annual consultant volume		% risk reduction
	0 procedures /year	40 procedures /year	
30-day Serious Adverse Events	1.16	0.74	35.9
90-day Serious Adverse Events	1.13	0.78	31.4
Prolonged hospital stay	1.25	0.64	49.1
Reoperation within 12 months	1.32	0.57	56.6
